# CUDR promotes liver cancer stem cell growth through upregulating TERT and C-Myc

**DOI:** 10.18632/oncotarget.5805

**Published:** 2015-10-19

**Authors:** Hu Pu, Qidi Zheng, Haiyan Li, Mengying Wu, Jiahui An, Xin Gui, Tianming Li, Dongdong Lu

**Affiliations:** ^1^ School of Life Science and Technology, Tongji University, Shanghai 200092, China

**Keywords:** liver cancer stem cell, CUDR, PTEN

## Abstract

Cancer up-regulated drug resistant (CUDR) is a novel non-coding RNA gene. Herein, we demonstrate excessive CUDR cooperates with excessive CyclinD1 or PTEN depletion to accelerate liver cancer stem cells growth and liver stem cell malignant transformation in *vitro* and in *vivo*. Mechanistically, we reveal the decrease of PTEN in cells may lead to increase binding capacity of CUDR to CyclinD1. Therefore, CUDR-CyclinD1 complex loads onto the long noncoding RNA H19 promoter region that may lead to reduce the DNA methylation on H19 promoter region and then to enhance the H19 expression. Strikingly, the overexpression of H19 increases the binding of TERT to TERC and reduces the interplay between TERT with TERRA, thus enhancing the cell telomerase activity and extending the telomere length. On the other hand, insulator CTCF recruits the CUDR-CyclinD1 complx to form the composite CUDR-CyclinD1-insulator CTCF complex which occupancied on the C-myc gene promoter region, increasing the outcome of oncogene C-myc. Ultimately, excessive TERT and C-myc lead to liver cancer stem cell and hepatocyte-like stem cell malignant proliferation. To understand the novel functions of long noncoding RNA CUDR will help in the development of new liver cancer therapeutic and diagnostic approaches.

## INTRODUCTION

Cancer stem cell(CSC) population is a subset of cells capable of dictating invasion, metastasis, heterogeneity, and therapeutic resistance in tumours. CSCs can be defined as a population of cells present in tumours, which can undergo self-renewal and differentiation. The evidence supports the vital role of this subset of cells in initiation and maintenance of a tumour in addition to their capability to dictate invasion, metastasis, heterogeneity, and therapeutic resistance in tumours. It is clear that heterogeneity amongst tumours and within tumour subtypes renders it difficult to discover unique markers. The well-accepted cancer stem cell surface markers are CD44, CD24, CD133, CD166, EpCAM [1]. CD44 and CD24 have been used extensively in combination or with other putative markers to isolate CSCs from solid tumours [2, 3]. CD44 is considered a potential CSC marker in majority of cancers [4]. CD24 is another important marker whose prognostic value and significance remains controversy [5]. Moreover, CD44^+^/CD133^+^ cells were enriched with tumour-initiating characteristics [6]. As CD24 and CD133 are enriched within epithelial and differentiated cells, more elucidations may require to define potential marker combination [7].

Increasing evidence suggests that non-coding RNAs have multiple important roles in transcriptional regulation, and also contribute to the expansion of genome complexity. LncRNAs can regulate gene expression in many ways, including chromosome remodeling, transcription and post-transcriptional processing [8]. Cancer up-regulated drug resistant (Urothelial cancer associated 1, UCA1, CUDR) is a novel non-coding RNA gene, which plays a pivotal role in cancer progression. Patients with high CUDR expression had a significantly poorer prognosis than those with low CUDR expression. Moreover, CUDR was found to influence the proliferation, apoptosis and cell cycle progression of colorectal cancer (CRC) cells [9]. CUDR plays a positive role in cancer cell glucose metabolism through the cascade of mTOR-STAT3/miR143-HK2 [10]. CUDR is a direct target of CAPERα/TBX3 repression whose overexpression is sufficient to induce senescence. Intriguingly, CUDR sequesters hnRNPA1 and thus stabilizes CDKN2A-p16INK. Thus CAPERα/TBX3 and CUDR constitute a coordinated, reinforcing mechanism to regulate both CDKN2A-p16INK transcription and mRNA stability [11]. CUDR increases the cisplatin resistance of bladder cancer cells by enhancing the expression of Wnt6, and thus represents a potential target to overcome chemoresistance in bladder cancer [12, 13, 14]. Expression of CUDR lncRNA was enhanced in tongue squamous cell carcinoma (TSCC) and may play a role in tumor metastasis [15]. CUDR regulated cell cycle through CREB via PI3K-AKT dependent pathway in bladder cancer [16]. CUDR is an oncofetal gene, and its upregulation may be important for carcinogenesis.

PTEN protein acts as a phosphatase to dephosphorylate phosphatidylinositol (3,4,5)-trisphosphate (PtdIns (3,4,5)P_3_ or PIP_3_). PTEN specifically catalyses the dephosporylation of the 3` phosphate of the inositol ring in PIP_3_, resulting in the biphosphate product PIP_2_ (PtdIns(4,5)P2). This dephosphorylation is important because it results in inhibition of the AKT signaling pathway [17]. When the PTEN enzyme is functioning properly, it acts as part of a chemical pathway that signals cells to stop dividing and can cause cells to undergo programmed cell death. There is also evidence that the protein made by the PTEN gene may play a role in cell migration and adhesion of cells to surrounding tissues [18]. PTEN orthologs have been identified in most mammals for which complete genome PTEN is one of the most commonly lost tumor suppressors in human cancer; in fact, up to 70% of men with prostate cancer are estimated to have lost a copy of the *PTEN* gene at the time of diagnosis [19]. During tumor development, mutations and deletions of PTEN occur that inactivate its enzymatic activity leading to increased cell proliferation and reduced cell death. Frequent genetic inactivation of PTEN occurs in glioblastoma, endometrial cancer, and prostate cancer; and reduced expression is found in many other tumor types such as lung and breast cancer. PTEN deletion mutants have recently been shown to allow nerve regeneration in mice [20]. The competition between PTEN mRNA and other RNAs for shared microRNA molecules has emerged as one such mechanism. The competing endogenous RNA (ceRNA) partners of PTEN that have been identified so far. PTEN-centered ceRNA networks can contribute to a deeper understanding of PTEN function and tumorigenesis [21].

CyclinD1 is characterized by a dramatic periodicity in protein abundance throughout the cell cycle. cyclinD1 forms a complex with and functions as a regulatory subunit of CDK4, whose activity is required for cell cycle G1/S transition. Evidence has established that members of the cyclin D1 family function to regulate phosphorylation of the retinoblastoma gene product, thereby activating E2F transcription factors. Blockage of NF-κB, STAT3, or cyclinD1 using siRNA transfection decreased the carcinogen-induced tumorigenesis in rats. Macrophage-initiated TNF-α/NF-κB/cyclinD1 and IL-6/STAT3/cyclinD1 pathways are primarily responsible for promoting lung tumorigenesis [22]. Flubendazole (widely used in the treatment of intestinal parasites) inhibited breast cancer cells proliferation in dose- and time-dependent manner and delayed tumor growth in xenograft models by intraperitoneal injection. Importantly, flubendazole reduced CD44 high/CD24low subpopulation and suppressed the formation of mammosphere and the expression of self-renewal related genes including c-myc, oct4, sox2, nanog and cyclinD1[23]. FOXO3 was vital in mediating doxorubicin-induced epithelial-mesenchymal transition (EMT). Activated FOXO3a disturbed the interaction between β-catenin and TCF and inhibited the expression of β-catenin/TCF target genes CyclinD1[24]. NTKL overexpression could accelerate the mitotic exit and chromosome segregation, which could promote G1/S transition by decreasing P53 and increasing CyclinD1 expressions [25].

In this report, our findings indicate overexpressed CUDR cooperates to overexpressed CyclinD1 or PTEN depletion to accelerate liver cancer stem cells and liver stem cells growth in *vitro* and in *vivo*. The abnormal CUDR-CyclinD1-PTEN-TERT/Myc axis leads to liver cancer stem cell and liver stem cells malignant transformation and proliferation.

## RESULTS

### CUDR cellular localization and transcriptional level in cancer stem cells, and human liver cancer stem cells isolation and its malignant growth capacity

**Figure 1:**
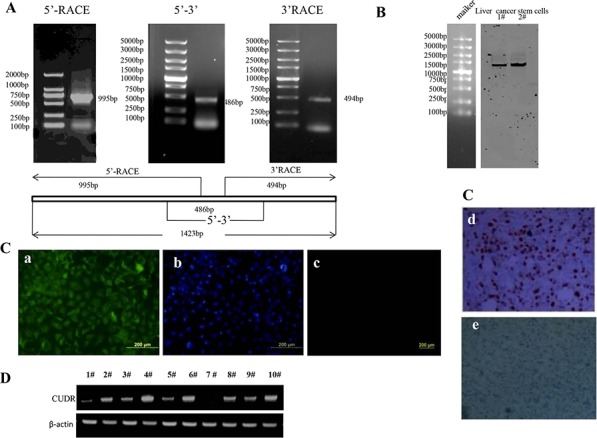

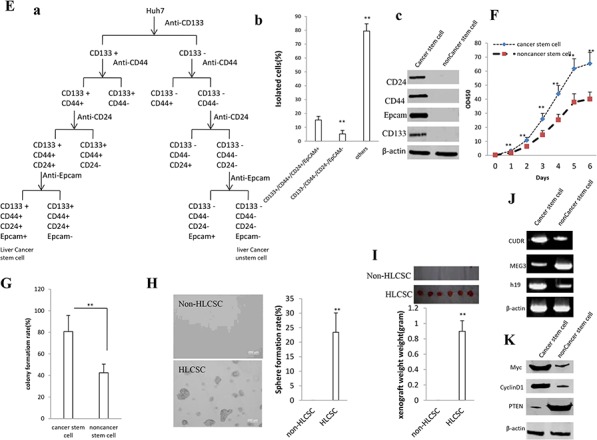
CUDR location and transcriptional level in cancer stem cells, and the comparsion of growth and gene expression between liver cancer stem cell and unstemic liver cancer cells **A.** CUDR cDNA full length analysis using 5′-RACE and 3′-RACE. **B.** Northern blotting analysis for CUDR in liver cancer stem cells. 1#. cell plasma; 2#. nucleus. **C.** *In situ* Hybridization for CUDR in liver cancer stem cells and liver cancer tissue. *a*. CUDR probe in liver cancer stem cells. *b*. DAPI staining in liver cancer stem cells. *c*. control unspecific probe in liver cancer stem cells. *d*. CUDR probe in liver cancer tissues. *e*. control unspecific probe in liver cancer tissues. **D.** RT-PCR analysis for CUDR transcriptional level in cancer unstem cells and cancer stem cells.β-actin as internal control. 1#. liver cancer unstem cells; 2#. liver cancer stem cell; 3#. breast cancer unstem cells; 4#. breast cancer stem cell; 5#. lung cancer unstem cells; 6#. lung cancer stem cell; 7#. leukaemia unstem cells; 8#. leukaemia stem cell; 9#. gastric cancer unstem cells; 9#. gastric cancer stem cell. **E.** Isolation and identification of liver cancer stem cells. *a*. The schematic digram illustrates a model of liver cancer stem cells isolated from human liver cancer cell line Huh7. *b*. Isolated cells from human liver cancer cell line Huh7 (CD133+/CD44+/CD24+/EpCAM+, CD133−/CD44−/CD24−/EpCAM− and others). Data are means of value from three independent experiment, bar ± SEM. **, *P* < 0.01; * *P* < 0.05. *c*. Western bloting with anti-CD24, anti-CD44, anti-EpCAM, anti-CD133 in liver cancer stem cell and unstemic liver cancer cell. **F.** Cell proliferation assay *in vitro* using CCK8 proliferation assay. Data are means of value from three independent experiment, bar ± SEM. ** *P* < 0.01; * *P* < 0.05. **G.** Cells colony-formation efficiency assay. Data are means of value from three independent experiment, bar ± SEM. ** *P* < 0.01; * *P* < 0.05. **H.** Cell sphere formation ability assay. Data are means of value from three independent experiment, bar ± SEM. ** *P* < 0.01; * *P* < 0.05. **I.** tumorigenesis tset *in vivo*. **J.** RT-PCR analysis of lncRNA CUDR, MEG3 and H19 in liver cancer stem cell and unstemic liver cancer cell. β-actin as internal control. **K.** Western blotting with anti-Myc, anti-CyclinD1 and anti-PTEN in liver cancer stem cell and unstemic liver cancer cell. β-actin as internal control.

To explore CUDR cellular localization and transcriptional level in cancer stem cells, we first analysed the CUDR cDNA full length using 5′-RACE and 3′-RACE. As shown in the Figure 1A, we found a 1423bp CUDR transcript at least in liver cancer stem cell. As well as we further identified the CUDR transcript size by Northern blotting analysis and showed CUDR was distributed in liver cancer stem cell plasma and nucleus respectively (Figure 1B). The findings of *In situ* Hybridization for CUDR either in liver cancer stem cells or in liver cancer tissues also showed CUDR was located in cell plasma and nucleus (Figure 1Ca–1Ce). Specifically, CUDR transcriptional level was significantly higher in cancer stem cells than in cancer unstem cells, including liver cancer, breast cancer, lung cancer, leukaemia and gastric cancer (Figure 1D).

To compare the growth and gene expression between liver cancer stem cell and unstemic liver cancer cells, we isolated the liver cancer stem cells from human liver cancer cell line Huh7 by CD133/CD44/CD24/EpCAM MicroBead according to the schematic digram (Figure 1Ea). In the isolated cells from human liver cancer cell line Huh7, Cells with CD133+/CD44+/CD24+/EpCAM+(HLCSC) was 15.3 ± 5.26%, Cells with CD133−/CD44−/CD24−/EpCAM-(non-HLCSC) was 5.23 ± 2.56% and others was 79.43 ± 5.19% (*P* < 0.01, respectively) (Figure 1Eb). We selected the CD133−/CD44− /CD24−/EpCAM− liver cancer cells as unstem cells (control cells). Although Epcam− cells as the nonstem cell population may exclude most cells with epithelial phenotype, these cells possess the lowest stemness. Western blotting showed that liver cancer stem cells CD133, CD44, CD24 and EpCAM were expressed in human liver cancer stem cells(HLCSC), as well as CD133, CD44, CD24 and EpCAM were not expressed in liver cancer unstem cells (non-HLCSC)(Figure 1Eb). Next, we examined cell proliferation ability, colony formation ability, sphere formation ability and tumor forming ability in immunodeficient mice in the two cell lines. As shown in Figure 1F, the growth rate was significantly increased in liver cancer stem cells compared to the liver cancer unstem cells (*P* < 0.01). As shown in Figure 1G, the colony formation rate in liver cancer stem cell group (80.7% ± 21.3%) was significantly higher than in liver cancer unstem cell group (42.5 ± 10.1%) (*P* < 0.01). HLCSCs possessed the higher sphere formation ability compared to non-LCSCs control (23.4 ± 6.7% vs 0%, *P* < 0.01) (Figure 1H). HLCSC produced the xenograft tumor in immunodeficient mice (0.898 ± 0.138 gram, *n* = 6, *p* < 0.01), on the constrary, non-HLCSC did not form xenograft tumor (*P* < 0.01)(Figure 1I). Further on, we detected the long noncoding RNA expression in the two cell lines by RT-PCR. As shown in Figure 1J, the CUDR and H19 expression were significantly increased in liver cancer stem cell line compared to the liver cancer unstem cell line. On the other hand, the MEG3 expression were significantly decreased in liver cancer stem cell line compared to the liver cancer unstem cell line. We detected the gene expression in the two cell lines by Western blotting. As shown in Figure 1K, the C-Myc and CyclinD1 expression were significantly increased in liver cancer stem cell line compared to the liver cancer unstem cell line, however, the PTEN expression were significantly decreased in liver cancer stem cell line compared to the liver cancer unstem cell line. Collectively, these observations suggests that CUDR may be associated with stem cell malignant transformation, and isolated human liver cancer stem cell possesses strong malignant growth capability and abnormal gene expression.

### The synergetic effect of long noncoding RNA CUDR, cyclinD1 and PTEN depletion promotes human liver cancer stem cell proliferation

**Figure 2:**
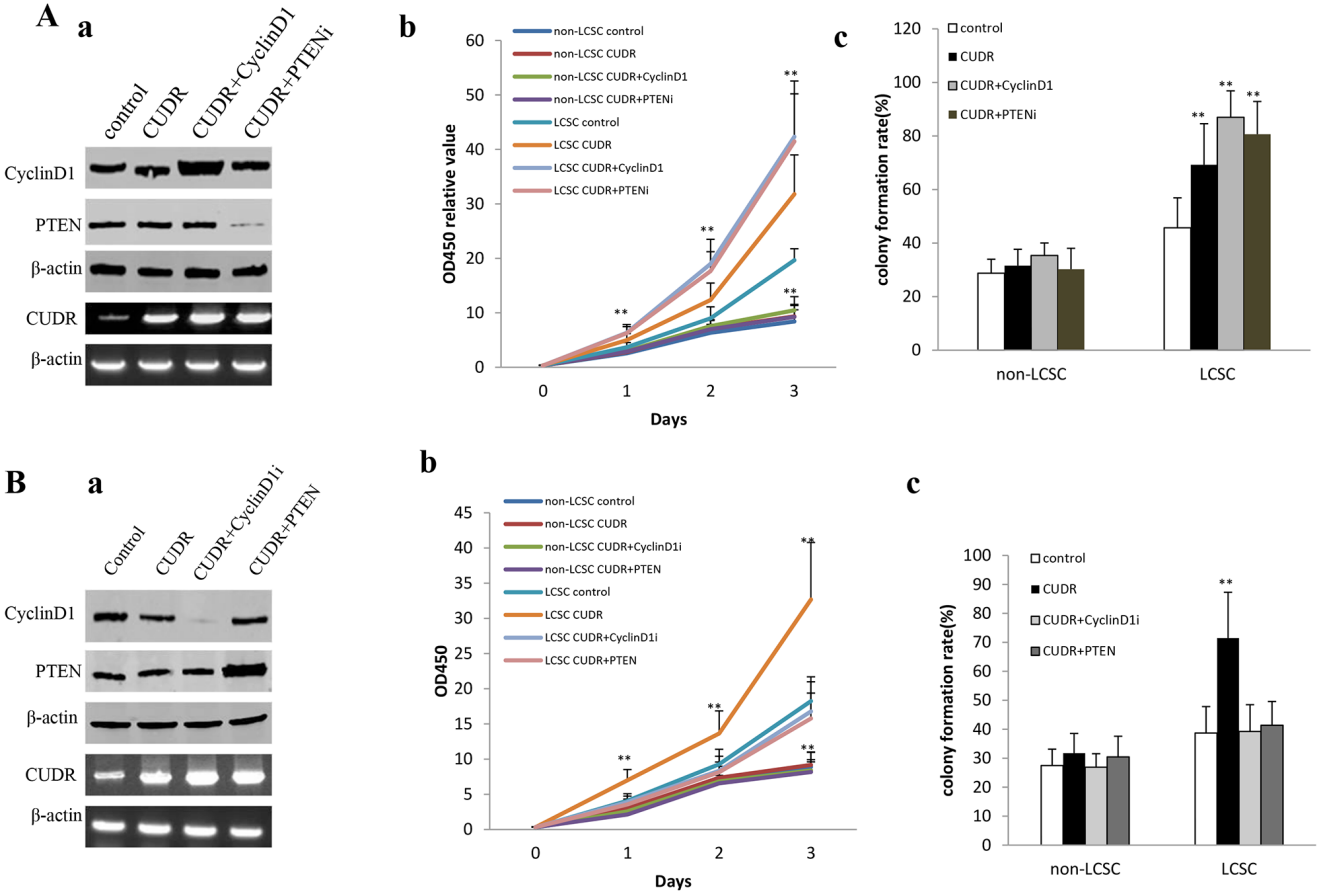

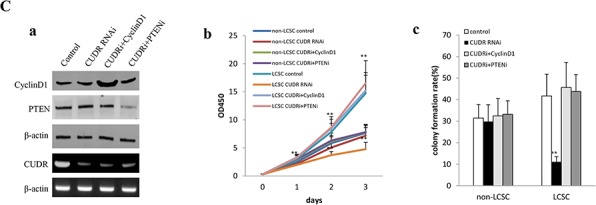
CUDR overexpression cooperated with cyclinD1 overexpression or PTEN depletion accerlates the liver cancer stem cell proliferation **A.** The growth and colony formation ability in the stable human liver cancer stem cell(HLCSC) lines and non-HLCSC transfected with pCMV6-A-GFP, pCMV6-A-GFP-CUDR, pCMV6-A-GFP-CUDR plus pcDNA3.1-CyclinD1, pCMV6-A-GFP-CUDR plus pGFP-V-RS-PTEN, respectively. *a*. RT-PCR analysis of CUDR mRNA and Western bloting with anti-cyclinD1, anti-PTEN expression in stable liver cancer stem cells transfected with pCMV6-A-GFP, pCMV6-A- GFP-CUDR, pCMV6-A-CUDR plus pcDNA3.1-CyclinD1, pCMV6-A- GFP-CUDR plus pGFP-V-RS-PTEN, respectively (indicated in the *left*). β-actin as internalcontrol. *b*. Cell proliferation assay *in vitro* in liver cancer stem cells and unstemic liver cancer. Data are means of value from three independent experiment, bar ± SEM. ** *P* < 0.01; * *P* < 0.05. c. Cells colony-formation efficiency assay in liver cancer stem cells and unstemic liver cancer cells. Data are means of value from three independent experiment, bar ± SEM. ** *P* < 0.01; * *P* < 0.05. **B.** The growth and colony formation ability in the stable human liver cancer stem cell (HLCSC) lines and non-HLCSC transfected with pCMV6-A-GFP, pCMV6-A-GFP-CUDR, pCMV6-A-GFP-CUDR plus pGFP-V-RS—CyclinD1, pCMV6-A-GFP-CUDR plus pcDNA3.1-PTEN, respectively. *a*. RT-PCR analysis of CUDR mRNA and Western bloting with anti-cyclinD1, anti-PTEN expression in stable liver cancer stem cells transfected with pCMV6-A-GFP, pCMV6-A-GFP-CUDR, pCMV6-A- GFP-CUDR plus pGFP-V-RS-CyclinD1, pCMV6-A-GFP-CUDR plus pcDNA3.1-PTEN, respectively (indicated in the *left*). β-actin as internalcontrol. *b*. Cell proliferation assay *in vitro* in liver cancer stem cells and unstemic liver cancer cells. Data are means of value from three independent experiment, bar ± SEM. ** *P* < 0.01; * *P* < 0.05. c. Cells colony-formation efficiency assay in liver cancer stem cells and unstemic liver cancer cell. Data are means of value from three independent experiment, bar ± SEM. ** *P* < 0.01; * *P* < 0.05. **C.** The growth and colony formation ability in the stable human liver cancer stem cell(HLCSC) lines and non-HLCSC transfected with pGFP-V-RS, pGFP-V-RS-CUDR, pGFP-V-RS-CUDR plus pcDNA3.1-CyclinD1, pGFP-V-RS-CUDR plus pGFP-V-RS-PTEN, respectively. *a*. RT-PCR analysis of CUDR mRNA and Western bloting with anti-cyclinD1, anti-PTEN expression in stable liver cancer stem cells transfected with pGFP-V-RS, pGFP-V-RS-CUDR, pGFP-V-RS-CUDR plus pcDNA3.1-CyclinD1, pGFP-V-RS-CUDR plus pGFP-V-RS-PTEN, respectively (indicated in the *left*). β-actin as internalcontrol. *b*. Cell proliferation assay *in vitro* in liver cancer stem cells and unstemic liver cancer cells. Data are means of value from three independent experiment, bar ± SEM. **, *P* < 0.01; *, *P* < 0.05. c. Cells colony-formation efficiency assay in liver cancer stem cells and liver cancer unstemic cells. Data are means of value from three independent experiment, bar ± SEM. ** *P* < 0.01; * *P* < 0.05.

To address whether CUDR overexpression cooperated with cyclinD1 overexpression or PTEN knockdown to accerlate the liver cancer stem cell proliferation, we established the stable human liver cancer stem cell(HLCSC) lines transfected with pCMV6-A-GFP, pCMV6-A-GFP-CUDR, pCMV6-A-GFP-CUDR plus pcDNA3.1-CyclinD1, pCMV6-A-GFP-CUDR plus pGFP-V-RS-PTEN, respectively. We confirmed CyclinD1 and PTEN expression using western blotting and the CUDR expression using RT-PCR. As shown in Figure 2Aa, the results showed that CyclinD1 was significantly overexpressed in pCMV6-A-GFP-CUDR plus pcDNA3.1-CyclinD1 transfected HLCSC cells compared to the control, as well as PTEN was significantly knocked down in pCMV6-A-GFP-CUDR plus pGFP-V-RS-PTEN transfected HLCSC cells compared to the control. CUDR was significantly overexpressed in pCMV6-A-GFP-CUDR, pCMV6-A-GFP-CUDR plus pcDNA3.1-CyclinD1, pCMV6-A-GFP-CUDR plus pGFP-V-RS-PTEN transfected HLCSC cells compared to the control. At the first time, we detected these cells proliferation *in vitro*. As shown in Figure 2Ab, CUDR overexpression, CUDR overexpression plus CyclinD1 overexpression, CUDR overexpression plus PTEN knockdown promoted the HLCSC proliferation compared to the control. Furthermore, CUDR overexpression plus CyclinD1 overexpression, CUDR overexpression plus PTEN knockdown added up to the greater degree. However, there is no significant difference among liver cancer unstem cells(non-HLCSC) groups transfected with pCMV6-A-GFP, pCMV6-AGFP-CUDR, pCMV6-A-GFP-CUDR plus pcDNA3.1-CyclinD1, pCMV6-A-GFP-CUDR plus pGFP-V-RS-PTEN respectively. Next, we conducted colony-formation efficiency assay in these liver cancer stem cells or liver cancer unstem cells. As shown in Figure 2Ac, the colony-formation rate added up to 69.26 ± 15.31%, 86.98 ± 9.89%, 80.67 ± 12.23% in CUDR overexpressed, CUDR overexpressed plus CyclinD1 overexpressed, CUDR overexpressed plus PTEN knocked-down HLCSC respectively, while the colony-formation was 45.67 ± 11.23% in control (*p* < 0.01). However, there was no significantly difference among CUDR overexpressed, CUDR overexpressed plus CyclinD1 overexpressed, CUDR overexpressed plus PTEN knocked-down and control liver cancer unstem cells (the colony-formation was 31.54 ± 6.12%, 35.34 ± 4.78%, 30.21 ± 7.81%, 28.76 ± 5.23%, *P* > 0.05, respectively).

To address whether CUDR overexpression cooperated with cyclinD1 knockdown or PTEN overexpression to influence on the liver cancer stem cell proliferation, we established the stable human liver cancer stem cell(HLCSC) lines transfected with pCMV6-A-GFP, pCMV6-A-GFP-CUDR, pCMV6-A-GFP-CUDR plus pGFP-V-RS—CyclinD1, pCMV6-A- GFP-CUDR plus pcDNA3.1-PTEN, respectively. We confirmed CyclinD1 and PTEN expression using Western blotting and the CUDR expression using RT-PCR. As shown in Figure 2Ba, the results showed that CyclinD1 was significantly knocked down in pCMV6-A-GFP-CUDR plus pGFP-V-RS-CyclinD1 transfected HLCSC cells compared to the control, as well as PTEN was significantly overexpressed in pCMV6-A-GFP-CUDR plus pcDNA3.1-PTEN transfected HLCSC cells compared to the control. CUDR was significantly overexpressed in pCMV6-A-GFP-CUDR, pCMV6-A-GFP-CUDR plus pGFP-V-RS—CyclinD1, pCMV6-A-GFP-CUDR plus pcDNA3.1-PTEN transfected HLCSC cells compared to the control. At the first time, we detected these cells proliferation *in vitro*. As shown in Figure 2Bb, CUDR overexpression promoted the HLCSC proliferation compared to the control (*P* < 0.01). On the other hand, CUDR overexpression plus CyclinD1 knockdown, CUDR overexpression plus PTEN overexpression did not alter cell proliferation ability compared to control (*P* > 0.05). Moreover, there is also no significant difference among liver cancer unstem cells groups transfected with pCMV6-A-GFP, pCMV6-A-GFP-CUDR, pCMV6-A- GFP-CUDR plus pGFP-V-RS-CyclinD1, pCMV6-A-GFP-CUDR plus pcDNA3.1-PTEN respectively (*P* > 0.05). Next, we performed colony-formation efficiency assay in these liver cancer stem cells or liver cancer unstem cells. As shown in Figure 2Bc, the colony-formation rate is significantly increased in CUDR overexpressed HLCSC compared to control (71.48 ± 15.78%, vs 38.71 ± 9.12%, *P* < 0.01), as well as the colony-formation rate in CUDR overexpressed plus CyclinD1 knocked down, CUDR overexpressed plus PTEN overexpressed HLCSC was not significantly altered compared to control (39.23 ± 9.23%, 41.35 ± 8.23% vs 38.71 ± 9.12%, *P* > 0.05, respectively). Moreover, there was no significantly difference among CUDR overexpressed, CUDR overexpressed plus CyclinD1 knocked-down, CUDR overexpressed plus PTEN overexpressed and control liver cancer unstem cells (the colony-formation was 31.74 ± 6.78%, 26.89 ± 4.67%, 30.45 ± 7.12%, 27.45 ± 5.67%, *P* > 0.05).

To address whether CUDR knockdown cooperated with cyclinD1 overexpression or PTEN knockdown to influence on the liver cancer stem cell proliferation, we established the stable human liver cancer stem cell(HLCSC) lines transfected with pGFP-V-RS, pGFP-V-RS-CUDR, pGFP-V-RS-CUDR plus pcDNA3.1-CyclinD1, pGFP-V-RS-CUDR plus pGFP-V-RS-PTEN, respectively. We confirmed CyclinD1 and PTEN expression using Western blotting and the CUDR expression using RT-PCR. As shown in Figure 2Ca, CyclinD1 was significantly overexptressed in pGFP-V-RS-CUDR plus pcDNA3.1-CyclinD1 transfected HLCSC cells compared to the control, as well as PTEN was significantly knocked down in pGFP-V-RS-CUDR plus pGFP-V-RS- PTEN transfected HLCSC cells compared to the control. CUDR was significantly knocked down in pGFP-V-RS-CUDR, pGFP-V-RS-CUDR plus pcDNA3.1-CyclinD1, pGFP-V-RS-CUDR plus pGFP-V-RS-PTEN transfected HLCSC cells compared to the control. We first detected these cells proliferation *in vitro*. As shown in Figure 2Cb, CUDR knockdown inhibited the HLCSC proliferation compared to the control (*P* < 0.01). On the other hand, CUDR knockdown plus CyclinD1 overexpression, CUDR knockdown plus PTEN knockdown did not alter cell proliferation capability compared to control (*P* > 0.05). However, there is no significant difference among liver cancer unstem cells groups transfected with pGFP-V-RS, pGFP-V-RS-CUDR, pGFP-V-RS-CUDR plus pcDNA3.1-CyclinD1, pGFP-V-RS-CUDR plus pGFP-V-RS-PTEN respectively (*P* > 0.05). Next, we performed colony-formation efficiency assay in these liver cancer stem cells or liver cancer unstem cells. As shown in Figure 2Cc, the colony-formation rate is significantly decreased in CUDR knocked-down HLCSC compared to control (11.12 ± 2.45% vs 41.71 ± 10.14%, *P* < 0.01). On the other hand, the colony-formation rate in CUDR knocked down plus CyclinD1 overexpressed, CUDR knocked-down plus PTEN knocked-down HLCSC was not significantly altered compared to control (45.67 ± 11.62%, 43.78 ± 7.91% vs 41.71 ± 10.14%, *P* > 0.05, respectively). However, there was no significantly difference among CUDR knocked down, CUDR knocked down plus CyclinD1 overexpressed, CUDR knocked-down plus PTEN knocked-down non-HLCSC and control liver cancer unstem cells (the colony-formation was 29.85 ± 7.82%, 32.46 ± 8.14%, 33.18 ± 6.39%, 31.45 ± 6.31%, *P* > 0.05). Taken together, these results suggest that excessive CUDR cooperates with excessive CyclinD1 or PTEN depletion to accelerate the liver cancer stem cells malignant proliferation.

### The synergetic effect of CUDR, CyclinD1 and PTEN depletion promotes human embroyic stem cell derived-hepatocyte-like cells growth and malignant transformation

**Figure 3:**
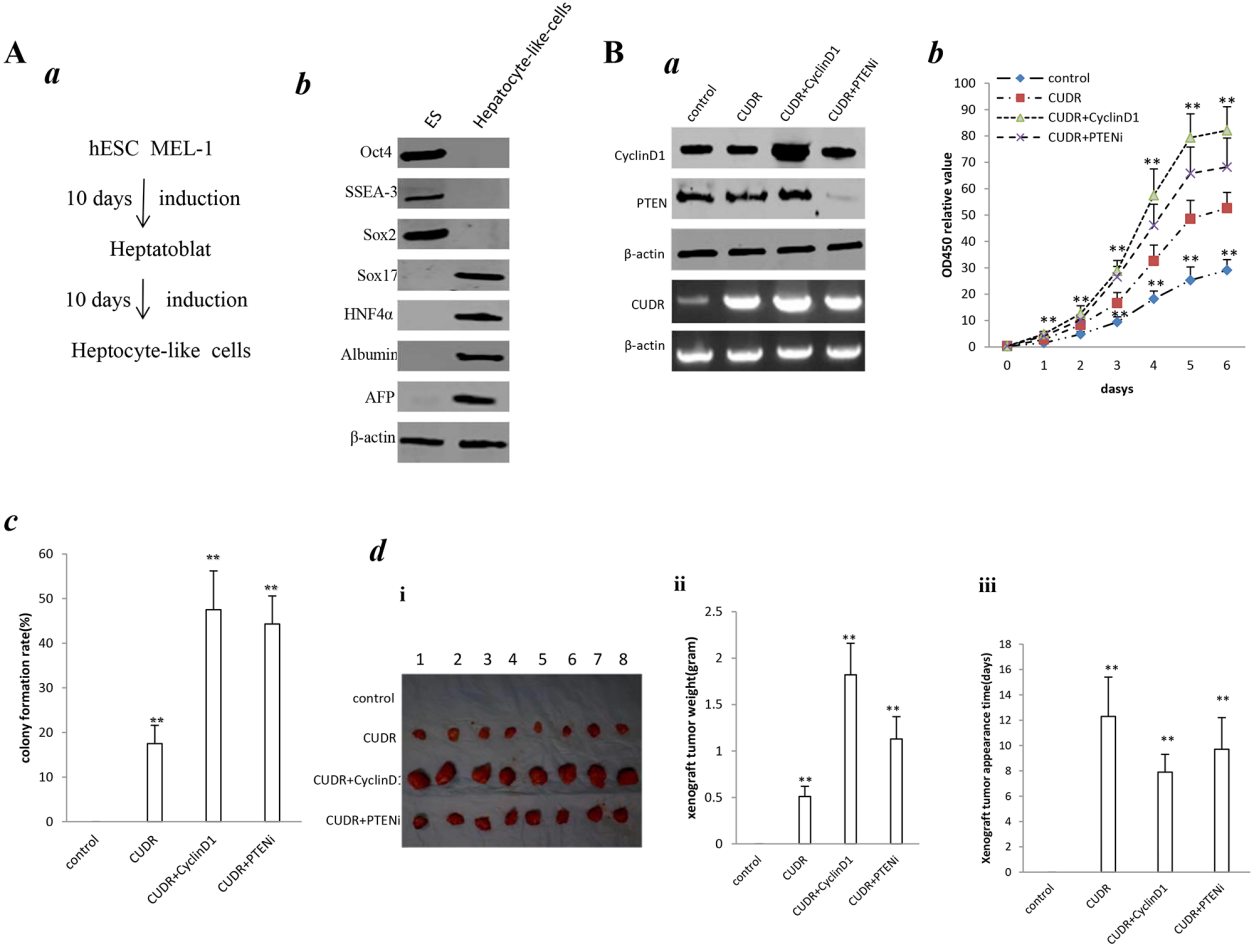

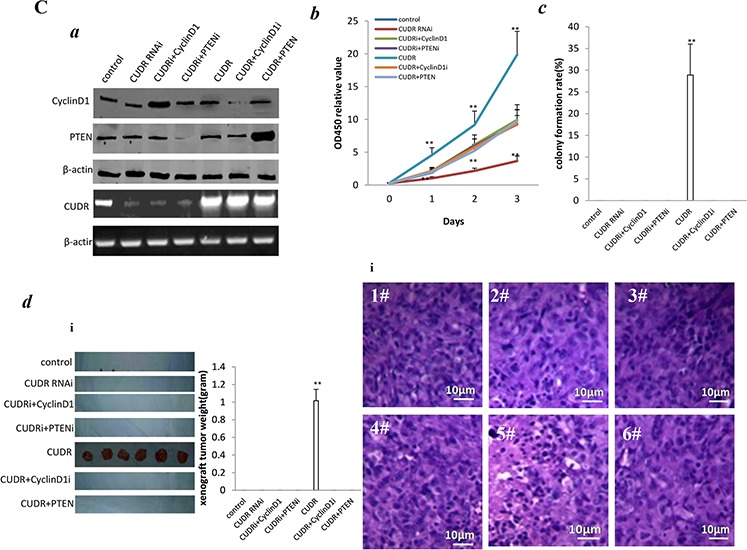
CUDR, cyclinD1 and PTEN synergistically alters induced hepatocyte-like cells growth *in vitro* and *in vivo* **A.** Induction and identification of hepatocyte-like cells. *a*. The schematic digram illustrates a model of liver stem cells induction from human embryic stem cells MEL-1. *b*. Western bloting with anti- Oct3, anti-SSEA3, anti-Sox2, anti-Sox17, anti-HNF4α, anti-Albumin, anti-AFP in liver stem cell and embryic stem cell MEL-1. β-actin as internal control. **B.** *a*. RT-PCR analysis of CUDR mRNA and Western bloting with anti-cyclinD1, anti-PTEN expression in stable hepatocyte-like cells transfected with pCMV6-A-GFP, pCMV6-A- GFP-CUDR, pCMV6-A-GFP-CUDR plus pcDNA3.1-CyclinD1, pCMV6-A-GFP-CUDR plus pGFP-V-RS PTEN, respectively (indicated in the *left*). β-actin as internal control. *b*. Cell proliferation assay *in vitro*. Data are means of value from three independent experiment, bar ± SEM. ** *P* < 0.01; * *P* < 0.05. *c*. Cells soft-agar colony-formation efficiency assay. Data are means of value from three independent experiment, bar ± SEM. ** *P* < 0.01; * *P* < 0.05. *d*. Tumorigenesis assay *in vivo*. The suspension of 5 × 108 (in 0.2 ml of PBS) hepatocyte-like cells transfected with pCMV6-A-GFP-CUDR, pcDNA3.1-CyclinD1, pGFP-V-RS-PTEN were injected subcutaneously at armpit in Balb/C mice. (*i*) The photography of xerograft tumors. (*ii*) Xenograft tumors weight in four groups indicated in figures. Data were means of value from eight Balb/C mice, mean ± SEM, *n* = 8, * *P* < 0.05; ** *P* < 0.01. *(iii)* Xenograft tumors onset time (days)in four groups. Data were means of value from eight SCID mice, mean ± SEM, *n* = 8, * *P* < 0.05; ** *P* < 0.01. **C.** *a*. RT-PCR analysis of CUDR mRNA and Western bloting with anti-cyclinD1, anti-PTEN in stable hepatocyte-like cells transfected with pGFP-V-RS, pGFP-V-RS-CUDR, pGFP-V-RS-CUDR plus pcDNA3.1-CyclinD1, pGFP-V-RS-CUDR plus pGFP-V-RS-PTEN, pCMV6-A-GFP-CUDR, pCMV6-A-GFP-CUDR plus pGFP-V-RS-CyclinD1, pCMV6-A-GFP-CUDR plus pcDNA3.1-PTEN, respectively (indicated in the *left*). β-actin as internal control. *b*. Cell proliferation assay *in vitro* Data are means of value from three independent experiment, bar ± SEM. **P < 0.01; *P < 0.05. *c*. Cells soft-agar colony-formation efficiency assay. Data are means of value from three independent experiment, bar ± SEM. **P < 0.01; *P < 0.05. *d*. Tumorigenesis assay *in vivo*. (*i*) The photography of xerograft tumors. (*ii*). Xenograft tumors weight in four groups. Data were means of value from eight Balb/C mice, mean ± SEM, *n* = 6, *P < 0.05; **P < 0.01. *(iii)* Xenograft tumors onset time (days)in four groups. Data were means of value from eight Balb/C mice, mean ± SEM, *n* = 6, *P < 0.05; **P < 0.01.

To assess CUDR, cyclinD1, PTEN synergistically affect on human embroyic stem cell derived-hepatocyte-like cells *in vitro* and *in vivo*, we first induced the hepatocyte-like cells from human embroyic stem cells MEL1 transfected with pCMV6-A-GFP, pCMV6-A-GFP-CUDR, pCMV6-A-GFP-CUDR plus pcDNA3.1-CyclinD1, pCMV6-A-GFP-CUDR plus pGFP-V-RS-PTEN, respectively. (Figure 3Aa). ES cells expressed Oct3, SSEA3, Sox2, as well as hepatocyte-like cells expressed Sox17, HNF4α, Albumin, AFP. It suggests we induced the hepatocyte-like cells from MEL-1 successfully (Figure 3Ab). As expected, CUDR was overexpressed in hepatocyte-like cells derived from MEL1 transfected with pCMV6-A-GFP-CUDR, pCMV6-A-GFP-CUDR plus pcDNA3.1-CyclinD1, pCMV6-A-GFP-CUDR plus pGFP-RS-GFP-PTEN. CyclinD1 was overexpressed in hepatocyte-like cells derived from MEL1 transfected with pCMV6-A-GFP-CUDR plus pcDNA3.1-CyclinD1. PTEN was knocked down in hepatocyte-like cells derived from MEL1 transfected with pCMV6-A-GFP-CUDR plus pGFP-RS-GFP-PTEN(Figure 3Ba). Cell proliferation assay showed CUDR overexpression, CUDR overexpression plus CyclinD1 overexpression and CUDR overexpression plus PTEN knockdown significantly promoted the growth of hepatocyte-like stem cells compared to control. Notably, CUDR overexpression plus CyclinD1 overexpression and CUDR overexpression plus PTEN knockdown made a greater extent promotion (Figure 3Bb). Notably, the soft-agar colony-formation efficiency rate was 17.5 ± 4.1%, 47.5 ± 8.7%%, 44.3 ± 6.3% in CUDR overexpression, CUDR overexpression plus CyclinD1 overexpression and CUDR overexpression plus PTEN knockdown group, while soft-agar colony-formation efficiency rate was 0% in control (*P* < 0.01, respectively). CUDR overexpression plus CyclinD1 overexpression and CUDR overexpression plus PTEN knockdown made a greater extent of colony-formation efficiency rate (Figure 3Bc). Next, we preformed tumorigenesis assay in *vivo*. As showed in Figure 3Bd(i&ii), the wet weight of xenograft tumors were significantly increased in CUDR overexpression, CUDR overexpression plus CyclinD1 overexpression and CUDR overexpression plus PTEN knockdown groups compared to control respectively (0.51 ± 0.11 gram, 1.82 ± 0.24 gram, 1.13 ± 0.34 gram vs 0, *P* < 0.01 respectively). Intriguingly, CUDR overexpression plus CyclinD1 overexpression and CUDR overexpression plus PTEN knockdown made a greater xenografts. In addition, the xenograft tumor appearance time (days) were significantly decreased in CUDR overexpression, CUDR overexpression plus CyclinD1 overexpression and CUDR overexpression plus PTEN knockdown groups compared to control respectively (12.3 ± 3.1 days, 7.9 ± 1.4 days, 9.7 ± 2.5 days vs 0 *P* < 0.01 respectively). Interestingly, CUDR overexpression plus CyclinD1 overexpression and CUDR overexpression plus PTEN knockdownpossessed a less xenografts onset time (Figure 3Bd(iii)). On the other hand, CUDR was knocked down in hepatocyte-like cells derived from MEL1 transfected with transfected with pGFP-V-RS—CUDR, pGFP-V-RS-CUDR plus pcDNA3.1-CyclinD1, pGFP-V-RS-CUDR plus pGFP-RS-GFP-PTEN. CUDR was overexpressed in hepatocyte-like cells derived from MEL1 transfected with pCMV6-A-GFP-CUDR, pCMV6-A-GFP-CUDR plus pGFP-V-RS-CyclinD1, pCMV6-A-GFP-CUDR plus pcDNA3.1-PTEN. CyclinD1 was overexpressed in hepatocyte-like cells derived from MEL1 transfected with transfected with pGFP-V-RS-CUDR plus pcDNA3.1-CyclinD1. CyclinD1 was knocked down in hepatocyte-like cells derived from MEL1 transfected with pCMV6-A-GFP-CUDR plus pGFP-V-RS-CyclinD1. PTEN was overexpressed in hepatocyte-like cells derived from MEL1 transfected with pCMV6-A-GFP-CUDR plus pcDNA3.1-PTEN. PTEN was knocked down in hepatocyte-like cells derived from MEL1 transfected with pGFP-V-RS-CUDR plus pGFP-RS-GFP-PTEN (Figure 3Ca). As expected, cell proliferation ability was increased only in CUDR overexpression group (*P* < 0.01) and decreased only in CUDR knockdown group (*P* < 0.01)(Figure 3Cb). Moreover, the soft-agar colonies were formed only in CUDR overexpression group (28.9 ± 7.12%) (Figure 3Cc) and the xenograft tumors were produced only in CUDR overexpression group (1.02 ± 0.13) (Figure 3Cd). Collectively, these results suggest the synergetic effect of long noncoding RNA CUDR overexpression plus CyclinD1 overexpression, CUDR overexpression plus PTEN depletion promotes human MEL1 derived-hepatocyte-like cells growth and malignant transformation.

### CUDR overexpression, CyclinD1 overexpression and PTEN knockdown synergistically enhance H19 expression in liver cancer stem cells

**Figure 4:**
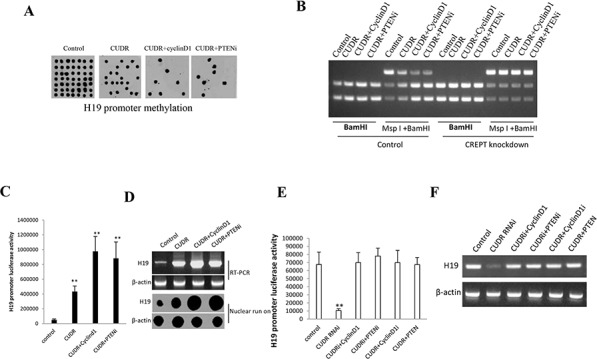
CUDR overexperssion cyclinD1 overexpression PTEN depletion synergistically enhances H19 expression on liver cancer stem cells **A.** H19 promoter methylation analysis by Methylated DNA Immunoprecipitation (MeDIP)-Dot blot-western blotting with anti-5-Methylcytosine (5-mC) in expression in stable liver cancer stem cells transfected with pCMV6-A-GFP, pCMV6-A- GFP-CUDR, pCMV6-A- GFP-CUDR plus pcDNA3.1-CyclinD1, pCMV6-A- GFP-CUDR plus pGFP-V-RS-PTEN, respectively (indicated in the *upper*). **B.** H19 promoter methylation analysis by MspI plus BamHI digestion in CREPT knockdown or control stable liver cancer stem cells transfected with pCMV6-A-GFP, pCMV6-A-GFP-CUDR, pCMV6-A-GFP-CUDR plus pcDNA3.1- CyclinD1, pCMV6-A-GFP-CUDR plus pGFP-V-RS-PTEN, respectively PTEN, respectively (indicated in the *upper and lower*). **C.** H19 promoter luciferase activity assay in in stable liver cancer stem cells transfected with pCMV6-A-GFP, pCMV6-A- GFP-CUDR, pCMV6-A-GFP-CUDR plus pcDNA3.1-CyclinD1, pCMV6-A- GFP-CUDR plus pGFP-V-RS PTEN, respectively. Each value was presented as mean ± standard error of the mean (SEM). **P < 0.01; *P < 0.05 **D.** H19 expression analysis by RT-PCR with H19 cDNA primers and Nuclear run on with Biotin-H19 probe. β-actin as internal control. **E.** H19 promoter luciferase activity assay in stable liver cancer stem cells transfected with pGFP-V-RS, pGFP-V-RS-CUDR, pGFP-V-RS-CUDR plus pcDNA3.1-CyclinD1, pGFP-V-RS-CUDR plus pGFP-V-RS PTEN, pCMV6-A-GFP-CUDR plus pGFP-V-RS-CyclinD1, pCMV6-A-GFP-CUDR plus pcDNA3.1-PTEN respectively. Each value was presented as mean ± standard error of the mean (SEM). **P < 0.01 ;*P < 0.05 **F.** H19 expression analysis by RT-PCR with H19 cDNA primers. β-actin as internal control.

To explore whether CUDR overexpression, cyclinD1 overexpression, PTEN knockdown synergistically impacted on H19 expression in liver cancer stem cells, we first performed H19 promoter methylation analysis by Methylated DNA Immunoprecipitation (MeDIP)-Dot blot-western blotting with anti-5-Methylcytosine (5-mC) in expression in stable liver cancer stem cells transfected with pCMV6-A-GFP, pCMV6-A-GFP-CUDR, pCMV6-A- GFP-CUDR plus pcDNA3.1-CyclinD1, pCMV6-A- GFP-CUDR plus pGFP-V-RS-PTEN, respectively. As shown in Figure 4A, CUDR overexpression, CUDR overexpression plus CyclinD1 overexpression, CUDR overexpression plus PTEN knockdown decreased the H19 promoter methylation. Moreover, CUDR overexpression plus CyclinD1 overexpression, CUDR overexpression plus PTEN knockdown results in a greater effenciency. The findings from H19 promoter methylation analysis by MspI plus BamHI digestion showed that CUDR overexpression, CUDR overexpression plus CyclinD1 overexpression, CUDR overexpression plus PTEN knockdown decreased the H19 promoter methylation. Moreover, CUDR overexpression plus CyclinD1 overexpression, CUDR overexpression plus PTEN knockdown results in a greater effenciency. However, CREPT (***c***ell-cycle ***r***elated and ***e***xpression- elevated ***p***rotein in ***t***umor) knockdown abrogated these actions (Figure 4B), suggesting CREPT may regulate the CUDR function. The luciferase activity assay results showed that CUDR overexpression, CUDR overexpression plus CyclinD1 overexpression, CUDR overexpression plus PTEN knockdown increased the H19 promoter luciferase activity. Morever, CUDR overexpression plus CyclinD1 overexpression, CUDR overexpression plus PTEN knockdown results in a greater effenciency (Figure 4C). RT-PCR and Nuclear run on results showed that CUDR overexpression, CUDR overexpression plus CyclinD1 overexpression, CUDR overexpression plus PTEN knockdown increased the H19 expresion. Morever, CUDR overexpression plus CyclinD1 overexpression, CUDR overexpression plus PTEN knockdown results in a greater effenciency (Figure 4D). The luciferase activity assay results showed that CUDR knockdown decreased the H19 promoter luciferase activity. CUDR knockdown plus CyclinD1 overexpression, CUDR knockdown plus PTEN knockdown, CUDR overexpression plus CyclinD1 knockdown, CUDR overexpression plus PTEN overexpression did not alter H19 promoter luciferase activity compared to control (Figure 4E). RT-PCR results showed that CUDR knockdown decreased the H19 transcription, however, H19 expression was not changed these groups of CUDR knockdown plus CyclinD1 overexpression, CUDR knockdown plus PTEN knockdown, CUDR overexpression plus CyclinD1 knockdown, CUDR overexpression plus PTEN overexpression compared to control (Figure 4F). Together, these results suggest CUDR overexpression cyclinD1 overexpression PTEN knockdown synergistically enhances H19 expression in liver cancer stem cells.

### CUDR, cyclinD1, PTEN collectively governs telomere through H19 in liver cancer stem cells

**Figure 5:**
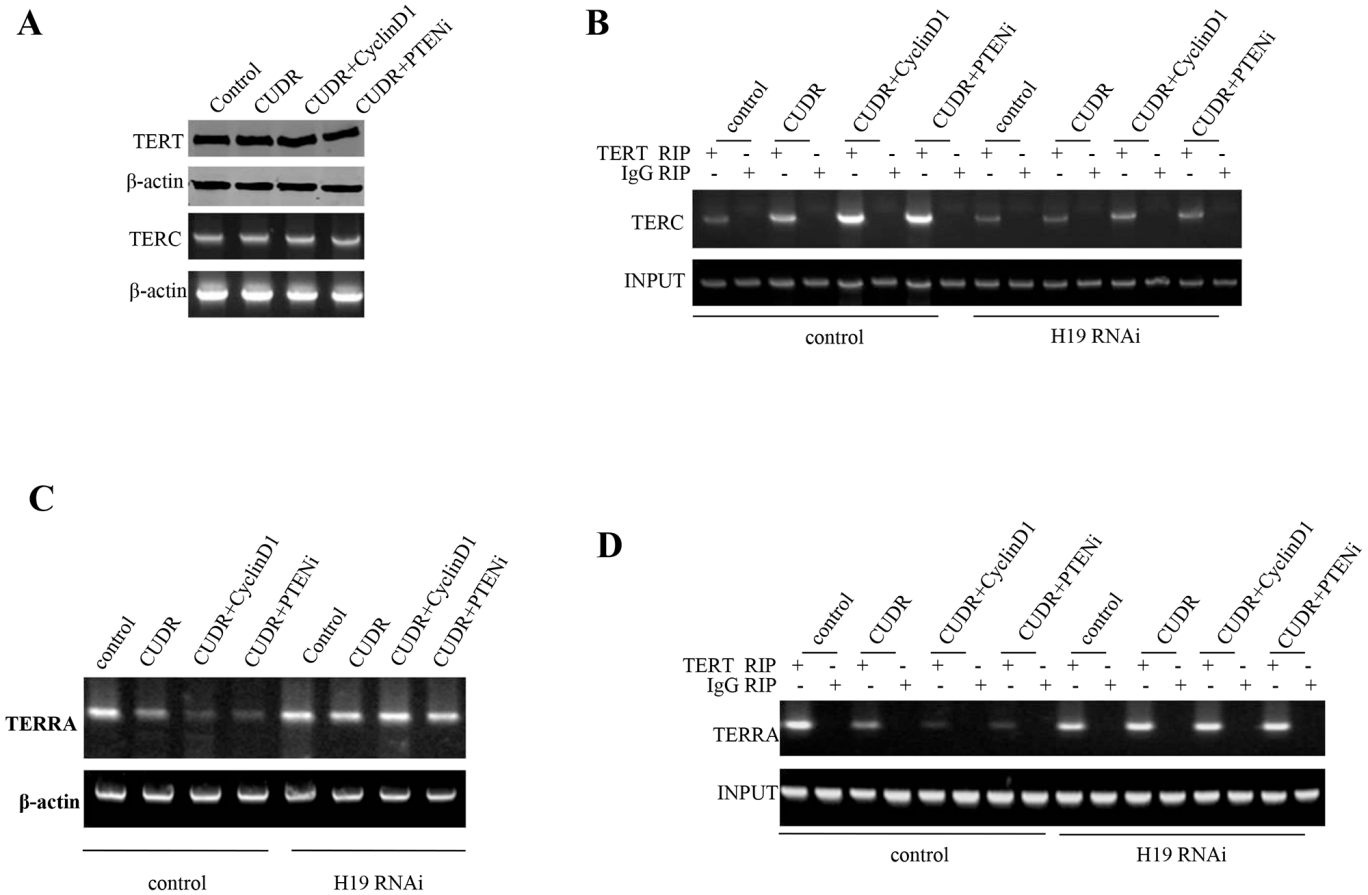

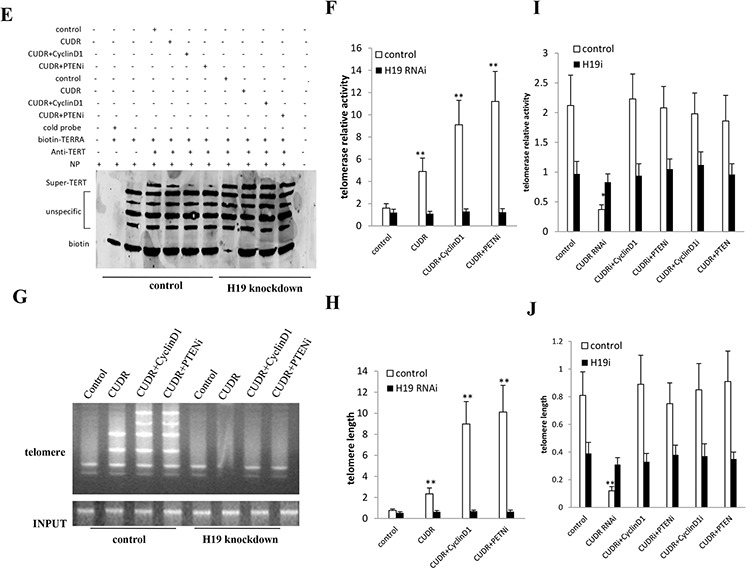
CUDR combined cyclinD1 or PTEN depletion collectly increases telomerase activity through H19 in human liver cancer stem cell **A**. RT-PCR analysis of TERC mRNA and Western blotting with anti-cyclinD1, anti-PTEN expression in stable liver cancer stem cells transfected with pCMV6-A-GFP, pCMV6-A-GFP-CUDR, pCMV6-A- GFP-CUDR plus pcDNA3.1-CyclinD1, pCMV6-A-GFP-CUDR plus pGFP-V-RS PTEN, respectively (indicated in the *left*). β-actin as internal control. **B.** RNA Immunoprecipitation(RIP) with anti-TERT followed by RT-PCR with TERC mRNA primers in contro or H19 knocked-down stable liver cancer stem cells transfected with pCMV6-A-GFP, pCMV6-A-GFP-CUDR, pCMV6-A-GFP-CUDR plus pcDNA3.1-CyclinD1, pCMV6-A-GFP-CUDR plus pGFP-V-RS PTEN, respectively. IgG RIP as negative controlTERC mRNA as INPUT. **C.** RT-PCR analysis of TERRA mRNA in control or H19 knocked-down stable liver cancer stem cells transfected with pCMV6-A-GFP, pCMV6-A-GFP-CUDR, pCMV6-A-GFP-CUDR plus pcDNA3.1-CyclinD1, pCMV6-A-GFP-CUDR plus pGFP-V-RS-PTEN, respectively. **D.** RNA Immunoprecipitation(RIP) with anti-TERT followed by RT-PCR with TERRA mRNA primers in contro or H19 knocked-down stable liver cancer stem cells transfected with pCMV6-A-GFP, pCMV6-A- GFP-CUDR, pCMV6-A-GFP-CUDR plus pcDNA3.1-CyclinD1, pCMV6-A- GFP-CUDR plus pGFP-V-RS PTEN, respectively. IgG RIP as negative control TERC mRNA as INPUT. **E.** Super-EMSA(gel-shift) with biotin- TERRA cRNA probe and anti-TERT antibody. The intensity of the band was examined by Western blotting with anti-Biotin. **F.** Telomerase activity assay with TRAP method mRNA in control or H19 knocked-down stable liver cancer stem cells transfected with pCMV6-A-GFP, pCMV6-A-GFP-CUDR, pCMV6-A-GFP-CUDR plus pcDNA3.1-CyclinD1, pCMV6-A- GFP-CUDR plus pGFP-V-RS PTEN, respectively. Each value was presented as mean ± standard error of the mean (SEM). **P < 0.01; *P < 0.05 **G.** The PCR detection of telomere repeat sequence in control or H19 knocked-down stable liver cancer stem cells transfected with pCMV6-A-GFP, pCMV6-A-GFP-CUDR, pCMV6-A-GFP-CUDR plus pcDNA3.1-CyclinD1, pCMV6-A-GFP-CUDR plus pGFP-V-RS PTEN, respectively. Each value was presented as mean ± standard error of the mean (SEM). **H.** The real-time PCR detection of telomere length in control or H19 knocked-down stable liver cancer stem cells transfected with pCMV6-A-GFP, pCMV6-A-GFP-CUDR, pCMV6-A-GFP-CUDR plus pcDNA3.1-CyclinD1, pCMV6-A-GFP-CUDR plus pGFP-V-RS PTEN, respectively. Each value was presented as mean ± standard error of the mean (SEM). **P < 0.01; *P < 0.05 **I.** Telomerase activity assay with TRAP method mRNA in control or H19 knocked-down stable liver cancer stem cells transfected with pGFP-V-RS, pGFP-V-RS-CUDR, pGFP-V-RS-CUDR plus pcDNA3.1-CyclinD1, pGFP-V-RS-CUDR plus pGFP-V-RS PTEN, pCMV6-A-GFP-CUDR plus pGFP-V-RS-CyclinD1, pCMV6-A-GFP-CUDR plus pcDNA3.1-PTEN respectively. Each value was presented as mean ± standard error of the mean (SEM). **P < 0.01; *P < 0.05 **J.** The real-time PCR detection of telomere length in control or H19 knocked-down stable liver cancer stem cells transfected with pGFP-V-RS, pGFP-V-RS-CUDR, pGFP-V-RS-CUDR plus pcDNA3.1-CyclinD1, pGFP-V-RS-CUDR plus pGFP-V-RS PTEN, pCMV6-A-GFP-CUDR plus pGFP-V-RS-CyclinD1, pCMV6-A-GFP-CUDR plus pcDNA3.1-PTEN respectively. Each value was presented as mean ± standard error of the mean (SEM). **P < 0.01; *P < 0.05.

To identity whether CUDR, cyclinD1, PTEN depletion synergistically altered the telomere activity through H19, we first constructed the stable liver cancer stem cell lines, including pCMV6-A-GFP, pCMV6-A-GFP-CUDR, pCMV6-A- GFP-CUDR plus pcDNA3.1-CyclinD1, pCMV6-A-GFP-CUDR plus pGFP-V-RS-PTEN, pCMV6-A-GFP plus pGFP-V-RS-H19, pCMV6-A-CUDR plus pGFP-V-RS-H19, pCMV6-A-GFP-CUDR plus pcDNA3.1-CyclinD1 plus pGFP-V-RS-H19, pCMV6-A-GFP-CUDR plus pGFP-V-RS PTEN plus pGFP-V-RS-H19. Our results showed that there was no significantly difference of TERT and TERC expression among these liver stem cell lines transfected with pCMV6-A-GFP, pCMV6-A- GFP-CUDR, pCMV6-A-GFP-CUDR plus pcDNA3.1-CyclinD1, pCMV6-A- GFP-CUDR plus pGFP-V-RS PTEN (Figure 5A). Intriguingly, CUDR overexpression, CUDR overexpression plus CyclinD1 overexpression and CUDR overexpression plus PTEN knockdown significantly enhanced the interplay between TERT and TERC compared to control. Notably, CUDR overexpression plus CyclinD1 overexpression and CUDR overexpression plus PTEN knockdown made a greater extent. However, this action was fully abrogated when H19 was knocked down in these liver stem cells (Figure 5B). Moreover, CUDR overexpression, CUDR overexpression plus CyclinD1 overexpression and CUDR overexpression plus PTEN knockdown significantly inhibited the long noncoding RNA TERRA expression compared to control. Notably, CUDR overexpression plus CyclinD1 overexpression and CUDR overexpression plus PTEN knockdown made a greater extent. However, this action was fully abrogated when H19 was knocked down in these liver stem cells (Figure 5C). Importantly, CUDR overexpression, CUDR overexpression plus CyclinD1 overexpression and CUDR overexpression plus PTEN knockdown significantly decreased the interplay between TERT and TERRA compared to control. Notably, CUDR overexpression plus CyclinD1 overexpression and CUDR overexpression plus PTEN knockdown made a greater extent. However, this action was fully abrogated when H19 was knocked down in these liver stem cells (Figure 5D). Super-EMSA(gel-shift) with biotin-TERRA cRNA probe and anti-TERT antibody findings showed that CUDR overexpression, CUDR overexpression plus CyclinD1 overexpression and CUDR overexpression plus PTEN knockdown significantly decreased the interaction between TERT and TERRA compared to control. Notably, CUDR overexpression plus CyclinD1 overexpression and CUDR overexpression plus PTEN knockdown made a greater extent. However, this action was fully abrogated when H19 was knocked down in these liver stem cells (Figure 5E). Telomerase activity assay with TRAP method showed that CUDR overexpression, CUDR overexpression plus CyclinD1 overexpression and CUDR overexpression plus PTEN knockdown significantly increased the TERT activity compared to control. Notably, CUDR overexpression plus CyclinD1 overexpression and CUDR overexpression plus PTEN knockdown made a greater extent. However, this action was fully abrogated when H19 was knocked down in these liver stem cells (Figure 5F). Both the PCR detection of telomere repeat sequence (Figure 5G) and The real-time PCR detection of telomere length (Figure 5H) showed that CUDR overexpression, CUDR overexpression plus CyclinD1 overexpression and CUDR overexpression plus PTEN knockdown significantly increased the telomere length compared to control. Notably, CUDR overexpression plus CyclinD1 overexpression and CUDR overexpression plus PTEN knockdown made a greater extent. However, this action was fully abrogated when H19 was knocked down in these liver stem cells. On the other hand, CUDR knockdown significantly decreased the TERT activity, while CUDR knockdown plus CyclinD1 overexpression, CUDR knockdown plus PTEN knockdown, CUDR overexpression plus CyclinD1 knockdown, CUDR overexpression plus PTEN overexpression did not alter the TERT activity compared to control (Figure 5I). CUDR knockdown significantly decreased the telomere length, as well as CUDR knockdown plus CyclinD1 overexpression, CUDR knockdown plus PTEN knockdown, CUDR overexpression plus CyclinD1 knockdown, CUDR overexpression plus PTEN overexpression did not alter the the telomere length compared to control (Figure 5J). Together, these observations strongly suggest CUDR combined cyclinD1 or PTEN knockdown collectly governs telomerase activity through H19 in liver cancer stem cell positively

### CUDR combined cyclinD1 or PTEN depletion collectively increases C-myc expression dependent on CTCF

**Figure 6:**
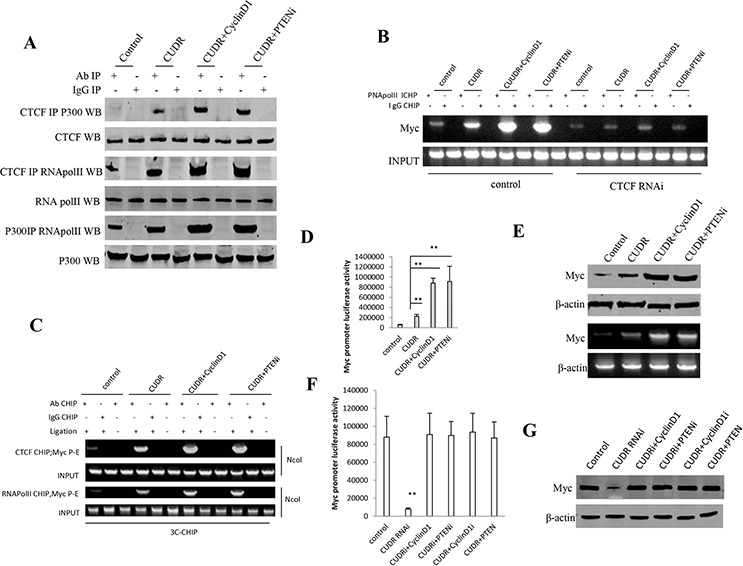
CUDR combined cyclinD1 or PTEN depletion collectly increases C-myc expression dependent on CTCF **A.** anti-CTCF or anti-P300 Co-Immunoprecipitation(IP) followed by Western blotting with anti-RNApolII, anti-P300, anti-CTCF expression in stable liver cancer stem cells transfected with pCMV6-A-GFP, pCMV6-A-GFP-CUDR, pCMV6-A-GFP-CUDR plus pcDNA3.1-CyclinD1, pCMV6-A-GFP-CUDR plus pGFP-V-RS PTEN, respectively. IgG IP as negative control. INPUT refers to Western blotting with anti-RNApolII, anti-P300, anti-CTCF. **B.** Chromatin Immunoprecipitation(CHIP) with anti-RNA PolII followed by PCR with C-myc promoter primer in control or CTCF knocked-down stable liver cancer stem cells transfected with pCMV6-A-GFP, pCMV6-A-GFP-CUDR, pCMV6-A-GFP-CUDR plus pcDNA3.1-CyclinD1, pCMV6-A-GFP-CUDR plus pGFP-V-RS PTEN, respectively. IgG CHIP as negative control. C-myc promoter DNA as INPUT. **C.** Chromosome conformation capture (3C) -chromatin immunoprecipitation (ChIP) with anti-CTCF, anti-RNA polII in stable liver cancer stem cells transfected with pCMV6-A-GFP, pCMV6-A-GFP-CUDR, pCMV6-A-GFP-CUDR plus pcDNA3.1-CyclinD1, pCMV6-A-GFP-CUDR plus pGFP-V-RS PTEN, respectively. The chromatin is cross-linked, digested with restriction enzymes, and ligated under conditions that favor intramolecular ligation. Immediately after ligation, the chromatin is immunoprecipitated using an antibody (anti-CTCF, anti-RNA polII)against the protein of interest. Thereafter, the cross-links are reversed, and the DNA is purified further. The PCR anlysis is applied for detecting c-myc promoter-enhancer coupling product using C-myc promoter and enhancer primers. The C-myc promoter and enhancer as INPUT. **D.** C-myc promoter luciferase activity assay in stable liver cancer stem cells transfected with pCMV6-A-GFP, pCMV6-A-GFP-CUDR, pCMV6-A-GFP-CUDR plus pcDNA3.1-CyclinD1, pCMV6-A-GFP-CUDR plus pGFP-V-RS PTEN, respectively. Each value was presented as mean ± standard error of the mean (SEM). **P < 0.01; *P < 0.05. **E.** RT-PCR analysis of C-myc mRNA and Western blotting with anti-C-myc expression in stable liver stem cells transfected with pCMV6-A-GFP, pCMV6-A-GFP-CUDR, pCMV6-A-GFP-CUDR plus pcDNA3.1-CyclinD1, pCMV6-A-GFP-CUDR plus pGFP-V-RS PTEN, respectively (indicated in the *left*). β-actin as internal control. **F.** C-myc promoter luciferase activity assay in stable liver cancer stem cells transfected with pGFP-V-RS, pGFP-V-RS-CUDR, pGFP-V-RS-CUDR plus pcDNA3.1-CyclinD1, pGFP-V-RS-CUDR plus pGFP-V-RS-PTEN, pCMV6-A-GFP-CUDR plus pGFP-V-RS-CyclinD1, pCMV6-A-GFP-CUDR plus pcDNA3.1-PTEN respectively. Each value was presented as mean ± standard error of the mean (SEM). **P < 0.01; *P < 0.05. **G.** Western blotting with anti-C-myc expression in stable liver stem cells transfected with pGFP-V-RS, pGFP-V-RS-CUDR, pGFP-V-RS-CUDR plus pcDNA3.1-CyclinD1, pGFP-V-RS-CUDR plus pGFP-V-RS-PTEN, pCMV6-A-GFP-CUDR plus pGFP-V-RS-CyclinD1, pCMV6-A-GFP-CUDR plus pcDNA3.1-PTEN respectively. (indicated in the *left*). β-actin as internal control.

To address whether CUDR combined cyclinD1 or PTEN knockdown collectively alters C-myc expression, we first performed the Co-Immunoprecipitation(IP) in stable liver cancer stem cells transfected with pCMV6-A-GFP, pCMV6-A- GFP-CUDR, pCMV6-A-GFP-CUDR plus pcDNA3.1-CyclinD1, pCMV6-A- GFP-CUDR plus pGFP-V-RS PTEN, respectively. As showed in Figure 6A, CUDR overexpression, CUDR overexpression plus CyclinD1 overexpression and CUDR overexpression plus PTEN knockdown significantly increased the interaction among RNApolII, P300 and CTCF compared to control. Intriguingly, CUDR overexpression plus CyclinD1 overexpression and CUDR overexpression plus PTEN knockdown made a greater extent. Chromatin Immunoprecipitation(CHIP) results showed that CUDR overexpression, CUDR overexpression plus CyclinD1 overexpression and CUDR overexpression plus PTEN knockdown significantly increased the the loading of RNA polII onto the C-myc promoter region compared to control. Notably, CUDR overexpression plus CyclinD1 overexpression and CUDR overexpression plus PTEN knockdown made a greater extent. However, this action was fully abrogated when CTCF was knocked down in these liver stem cells (Figure 6B). Chromosome conformation capture (3C)-chromatin immunoprecipitation (ChIP) results showed that CUDR overexpression, CUDR overexpression plus CyclinD1 overexpression and CUDR overexpression plus PTEN knockdown significantly increased the CTCF, RNA polII entering the C-myc promoter-enhancer loop compared to control. Notably, CUDR overexpression plus CyclinD1 overexpression and CUDR overexpression plus PTEN knockdown made a greater extent (Figure 6C). Luciferase activity assay showed CUDR overexpression, CUDR overexpression plus CyclinD1 overexpression and CUDR overexpression plus PTEN knockdown significantly increased the C-myc promoter luciferase activity compared to control. Notably, CUDR overexpression plus CyclinD1 overexpression and CUDR overexpression plus PTEN knockdown made a greater extent (Figure 6D). RT-PCR analysis and Western blotting showed CUDR overexpression, CUDR overexpression plus CyclinD1 overexpression and CUDR overexpression plus PTEN knockdown significantly increased the C-myc transcription and translation compared to control. Notably, CUDR overexpression plus CyclinD1 overexpression and CUDR overexpression plus PTEN knockdown made a greater extent (Figure 6E). On the other hand, CUDR knockdown significantly decreased the C-myc promoter luciferase activity compared to control, while CUDR knockdown plus CyclinD1 overexpression, CUDR knockdown plus PTEN knockdown, CUDR overexpression plus CyclinD1 knockdown, CUDR overexpression plus PTEN overexpression did not alter the C-myc promoter luciferase activity compared to control (Figure 6F). CUDR knockdown significantly decreased the C-myc expression compared to control, as well as CUDR knockdown plus CyclinD1 overexpression, CUDR knockdown plus PTEN knockdown, CUDR overexpression plus CyclinD1 knockdown, CUDR overexpression plus PTEN overexpression did not alter the expression compared to control (Figure 6G). Collectively, the observations suggest that CUDR combined cyclinD1 or PTEN knockdown collectively increases C-myc expression dependent on CTCF.

### TERT and C-myc activity is crucial for the synergetic oncogenic effect of CUDR, CyclinD1 and PTEN knockdown

**Figure 7:**
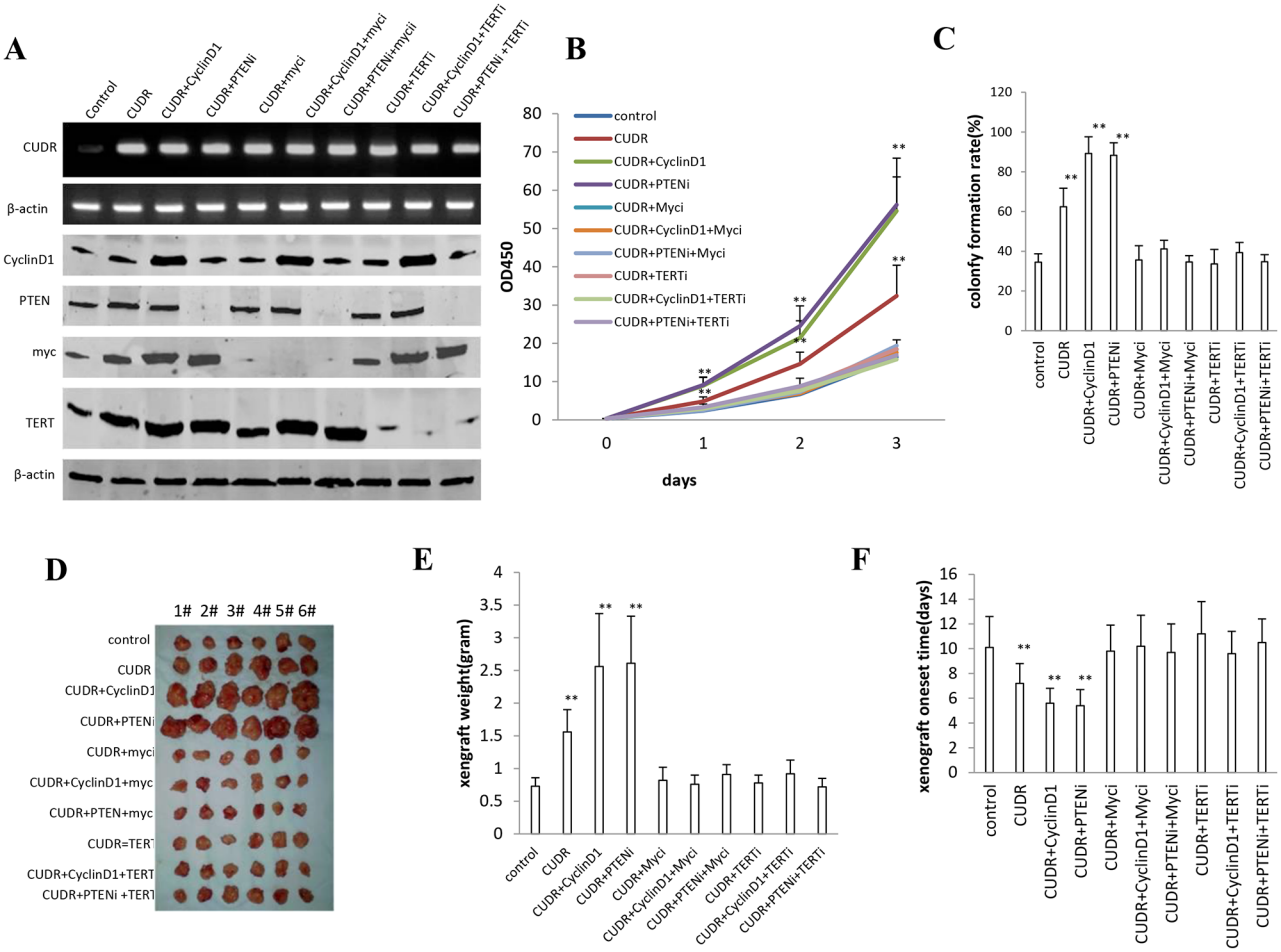
The rescued experiment of carcinogenesis effect of the synergetic effect of CUDR, CyclinD1 and PTEN knockdown C-myc knockdown or TERT knockdown abrogated the oncogenic function of CUDR combined with CyclinD1 or PTEN knockdown in stable liver cancer stem cells transfected with pCMV6-A-GFP, pCMV6-A-GFP-CUDR, pCMV6-A-GFP-CUDR plus pcDNA3.1-CyclinD1, pCMV6-A-GFP-CUDR plus pGFP-V-RS PTEN, pCMV6-A-CUDR plus pGFP-V-RS-C-myc, pCMV6-A-CUDR plus pcDNA3.1-CyclinD1 plus pGFP-V-RS-C-myc, pCMV6-A-GFP-CUDR plus pGFP-V-RS-PTEN plus pGFP-V-RS-C-myc, pCMV6-A-GFP-CUDR plus pGFP-V-RS-TERT, pCMV6-A-GFP-CUDR plus pcDNA3.1-CyclinD1 plus pGFP-V-RS-TERT, pCMV6-A-GFP-CUDR plus pGFP-V-RS-PTEN plus pGFP-V-RS- TERT, **A.** The RT-PCR of CUDR mRNA and the western blotting analysis with anti-CyclinD1, anti-PTEN, anti-C-myc and anti-TERT. β-actin as internal control. **B.** Cells growth assay. Each value was presented as mean ± standard error of the mean (SEM). **C.** Cells soft agar colony formation assay. Each value was presented as mean ± standard error of the mean (SEM). **P < 0.01; *P < 0.05. **D.** *In vivo* test in stable liver cancer stem cells transfected with pCMV6-A-GFP, pCMV6-A-GFP-CUDR, pCMV6-A-GFP-CUDR plus pcDNA3.1-CyclinD1, pCMV6-A-GFP-CUDR plus pGFP-V-RS PTEN, pCMV6-A-GFP-CUDR plus pGFP-V-RS-C-myc, pCMV6-A-GFP-CUDR plus pcDNA3.1-CyclinD1 plus pGFP-V-RS-C-myc, pCMV6-A-GFP-CUDR plus pGFP-V-RS PTEN plus pGFP-V-RS-C-myc, pCMV6-A-GFP-CUDR plus pGFP-V-RS-TERT, pCMV6-A-GFP-CUDR plus pcDNA3.1-CyclinD1 plus pGFP-V-RS-TERT, pCMV6-A-GFP-CUDR plus pGFP-V-RS PTEN plus pGFP-V-RS –TERT. *a*. The mice were stratified and the tumors were recovered. The photography of xerograft tumor in the four groups (indicated in left). *b*. The wet weight of each tumor was determined for each mouse. Each value was presented as mean ± standard error of the mean (SEM). **P < 0.01; *P < 0.05. *c*. The Xenograft appearance time. Each value was presented as mean ± standard error of the mean (SEM). **P < 0.01; *P < 0.05.

To further confirm the synergetic oncogenic effect of CUDR, CyclinD1 and PTEN depletion is related to the TERT and C-myc, we performed the rescued experiment of carcinogenesis in stable liver cancer stem cells transfected with pCMV6-A-GFP, pCMV6-A-GFP-CUDR, pCMV6-A-GFP-CUDR plus pcDNA3.1-CyclinD1, pCMV6-A- GFP-CUDR plus pGFP-V-RS-PTEN, pCMV6-A- GFP-CUDR plus pGFP-V-RS-C-myc, pCMV6-A-GFP-CUDR plus pcDNA3.1-CyclinD1 plus pGFP-V-RS-C-myc, pCMV6-A-GFP-CUDR plus pGFP-V-RS PTEN plus pGFP-V-RS-C-myc, pCMV6-A-GFP-CUDR plus pGFP-V-RS—TERT, pCMV6-A-GFP-CUDR plus pcDNA3.1-CyclinD1 plus pGFP-V-RS-TERT, pCMV6-A-GFP-CUDR plus pGFP-V-RS-PTEN plus pGFP-V-RS-TERT. The RT-PCR results showed that CUDR was overexpressed in transfected groups compared to the control. As shown in Figure 7A, the western blotting analysis showed CyclinD1 was overexpressed and PTEN, MYC, TERT were respectively kinocked down, and RT-PCR results showed CUDR was overexressed in these transfected cell lines. Cell growth assay results indicated that CUDR, CUDR plus CyclinD1 and CUDR plus PTEN depletion result in the greater incrument of cells growth, however, this action was abrogated when C-Myc or TERT was knocked down in these cell lines (Figure 7B). Although cells colony formation ability was higher in the cell lines transfected with CUDR (62.4 ± 9.3%), CUDR plus CyclinD1 (89.2 ± 8.4%) and CUDR plus PTEN RNAi (88.3 ± 6.3%) compared to control (34.5 ± 4.2%, *P* < 0.01, respectively), this action was abrogated when MYC or TERT was knocked down in these cell lines ((Figure 7C). Further on, tumorigenesis test showed that CUDR (1.56 ± 0.34 gram, 7.2 ± 1.6 days), CUDR plus CyclinD1 (2.56 ± 0.81 gram, 5.6 ± 1.2 days) and CUDR plus PTEN RNAi (2.61 ± 0.72 gram, 5.4 ± 1.3 days) results in the greater xenograft tumors and the shorter xenograft oneset time compared to control (0.73 ± 0.13 gram, 10.1 ± 2.5 days, *P* < 0.01, respectively), however, this action was abrogated when MYC or TERT was knocked down in these cell lines (Figure 7D, 7E, 7F). Together, these observations suggest that TERT and C-myc activity is crucial for the synergetic oncogenic effect of CUDR overexpression plus CyclinD1 overexpression or CUDR overexpression plus PTEN knockdown.

## DISCUSSION

**Figure 8:**
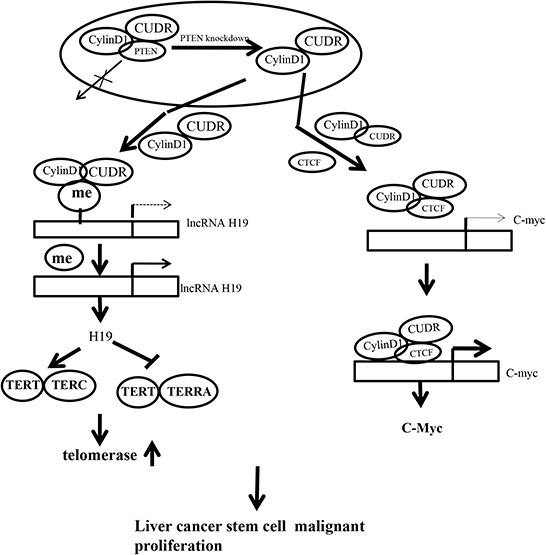
The schematic diagram illustrates a model that The synergetic effect of CUDR overexpression, CyclinD1 overexpression and PTEN depletion promotes liver cancer stem cells and liver stem cells malignant transformation through upregulation of C-myc and TERT Overexpressed CUDR cooperates to overexpressed CyclinD1 or PTEN knockout to accelerate liver cancer stem cells growth *in vitro* and *in vivo*. Mechanistically, we reveal the decrease of PTEN in cells may lead to increase binding capacity of CUDR to CyclinD1. Therefore, CUDR-CyclinD1 complex loads onto the long noncoding RNA H19 promoter region that may lead to reduce the DNA methylation on H19 promoter region and then to enhance the H19 expression. The overexpression of H19 increases the binding of TERT to TERC, while reducing the combination of TERT with TERRA, thus enhancing the cell telomerase activity and extending the telomere length. On the other hand, insulator CTCF recruits the CUDR-CyclinD1 complx to form the composite CUDR-CyclinD1-insulator CTCF complex which loads onto the C-myc gene promoter region, increasing the outcome of oncogene C-myc. In short, excessive TERT and C-myc lead to liver cancer stem cells and liver stem cells malignant proliferation.

It is well known that long non-coding RNAs (lncRNAs) are emerging as a novel set of targets for miRNAs. Long non-coding RNA (lncRNAs) played important roles in proliferation, apoptosis and invasiveness of tumor cells, and participated in metastatic capacity of cancers. In addition to regulating transcription, lncRNAs also control various aspects of post-transcriptional mRNA processing [26]. Our studies are now indicated to evaluate the effects of CUDR combined with CyclinD1 and PTEN depletion in liver cancer stem or liver stem cells (figure 8). Our present findings clearly demonstrate that overexpressed CUDR cooperates to overexpressed CyclinD1 or PTEN depletion to accelerate liver cancer stem cells, liver stem cells malignant transformation and growth in *vitro* and in *vivo*. The synergetic effect of CUDR, CyclinD1 and PTEN depletion is partly based on the upregulation of C-myc and TERT. Obviously, this is a new linkage of CUDR-CyclinD1-PTEN-TERT/C-myc in human liver cancer stem cells or liver stem cells.

It is worth mentioning that CUDR is a oncogenic long noncoding RNA and play an important role in the occurrence of hepatocellular carcinoma. In this report, we focused mainly on the view that CUDR plus CyclinD1 or CUDR plus PTEN depletion results in stronger oncogenic function in liver cancer stem cells and liver stem cells. Actually, our observations are consistent with these previous reports. For examples, CUDR promotes human bladder cancer cell proliferation and regulated cell cycle through CREB via PI3K-AKT dependent pathway in bladder cancer [27]. Herein, our results showed that CUDR combined with CyclinD1 or PTEN promotes liver cancer stem cells, liver stem cells malignant transformation and growth. The involvement of CUDR promotion of liver cancer cell growth is supported by results from six parallel sets of experiments: (1) CUDR overexpressed human liver cancer stem cells possess strong malignant growth ability. (2) Mechanistically, we reveal the decrease of PTEN in cancer cells may lead to increase binding capacity of CUDR to CyclinD1. (3) Therefore, CUDR-CyclinD1 complex loads onto the long noncoding RNA H19 promoter region that may lead to reduce the DNA methylation on H19 promoter region and then to enhance the H19 high expression. (4) Intriguingly, the overexpression of H19 increases the binding of TERT to TERC, while reducing the combination of TERT with TERRA, thus enhancing the cell telomerase activity and extending the telomere length. (5) On the other hand, insulator CTCF recruits the CUDR-CyclinD1 complx to form the composite CUDR-CyclinD1- insulator CTCF complex which loads onto the C-myc gene promoter region, increasing the outcome of oncogene C-myc. (6) Ultimatly, excessive TERT and C-myc lead to liver cancer stem cell malignant transformation and proliferation. Strikingly, although CUDR promotes liver stem cell malignant transformation only, CUDR plus CyclinD1 or CUDR plus PTEN depletion results in a stronger oncogenic function. We confer PTEN may inhibits CUDR cooperation with CyclinD1. Evidently, it is the results that the synergetic effect of long noncoding RNA CUDR, CyclinD1 and PTEN depletion promotes human liver cancer stem cell proliferation and triggers human liver stem cell growth and malignant transformation. According to the aforementioned findings and reorts, it is thus clear that CUDR overexpression combined with CyclinD1 overexpression and PTEN knockdown possesses a strong carcinogenic ability.

These findings are noteworthy that CUDR, cyclinD1, PTEN depletion synergistically enhances H19 expression. There is plenty of evidence that H19 acts as an oncogene. H19 is expressed at high levels in adrenocortical neoplasms, choriocarcinomas, hepatocellular carcinomas, bladder cancers, ovarian serous epithelial cancers, head and neck carcinomas, endometrial cancer, breast cancer, acute T cell leukemia/lymphoma, Wilms' tumor, testicular germ cell cancer, esophageal cancer and lung cancer [28, 29, 30, 31]. Overexpression of H19 appears to be important in the development of breast cacer, liver cancer, lung cancer, gastric cancer, esophageal and colorectal cancer cells [32, 33, 34, 35, 36]. The epigenetic regulation of imprinted genes by monoallelic DNA methylation of either maternal or paternal alleles is critical for embryonic growth and development [37]. Moreover, Histone H1.3 overexpression leads to increased occupancy of H1.3 at the H19 regulator region encompassing the imprinting control region (ICR), concomitant with increased DNA methylation and reduced occupancy of the insulator protein CTCF at the ICR. H1.3 dramatically inhibits H19 expression, which contributes to the suppression of epithelial ovarian carcinogenesis [38]. Although the increment of H19 may partly contribute to CUDR medicated promotion of liver cancer stem cells, liver stem cells growth, our findings in this study provide novel evidence for an active role of H19. This assertion is based on several observations: (1) CUDR overexpression, cyclinD1 overexpression, PTEN depletion collectively govern telomere activity and length through H19 in liver stem cells. (2) H19 promotes the interplay between TERT and TERC and reduce the interplay between TERT and TERRA which activated the telomerase and increased the telomere length. (3) TERT knockdown abrogated the oncogenic function of CUDR combined with CyclinD1 or CUDR combined with PTEN depletion. (4) TERT and C-myc activity is crucial for the synergetic oncogenic effect of CUDR, CyclinD1 and PTEN depletion.

It has been confirmed that HCCs expressing “stemness”-related proteins are characterized by increased telomere length, increased expression of hTERT and shelterin complex proteins, and increased chromosomal instability compared to conventional HCCs [39]. The telomeric long noncoding RNA Telomeric repeat-containing RNA (TERRA) has been implicated in modulating the structure and processing of deprotected telomeres. TERRA upregulation is occurring upon depletion of TRF2 at all transcribed telomeres. TERRA associates with SUV39H1 H3K9 histone methyltransferase, which promotes accumulation of H3K9me3 at damaged telomeres and end-to-end fusions [40]. TERRA is important for telomere regulation. TERRA G-quadruplex structure is critical for binding to telomeres [41]. Strikingly, a repression is observed on TRF2 through the binding of a TERRA-like RNA molecule to the N-terminus of TRF2[42].

On the other hand, our data suggest that CUDR combined cyclinD1 or PTEN depletion collectively increases C-myc expression dependent on CTCF. Cohesin co-localizes with CCCTC binding factor (CTCF), a zinc finger protein implicated in multiple gene regulatory events. At the imprinted IGF2-H19 locus, CTCF plays an important role in organizing allele-specific higher-order chromatin conformation and functions as an enhancer blocking transcriptional insulator. Cohesin-dependent, higher-order chromatin conformation of the locus exists in both G1 and G2 phases of the cell cycle and is therefore independent of cohesin's function in sister chromatid cohesion [43]. Current epigenomics approaches have facilitated the genome-wide identification of regulatory elements based on chromatin features and transcriptional regulator binding and have begun to map long-range interactions between regulatory elements and their targets. Species-specific transposable elements may influence such interactions by remodeling the CTCF binding repertoire [44, 45]. Some findings indicate that CTCF and cohesin are integral components of most human subtelomeres, and important for the regulation of TERRA transcription and telomere end protection [46]. A role for CTCF and cohesin in subtelomere chromatin organization, TERRA transcription, and telomere end protection [46]. CTCF binds to multiple imprinted loci and is required for proper imprinted expression at the H19/Igf2 locus [47]. It is evident that activation of C-Myc may play an important role in CUDR oncogenic action in liver cancer stem cells. Our findings in this study provide novel evidence for an active role of C-myc in CUDR-mediated promotion of liver cancer stem cell growth. This assertion is based on several observations: (1) CUDR combined with CyclinD1, CUDR combined with PTEN depletion enhanced the C-myc expression; (2) C-myc knockdown abrogated the oncogenic function of CUDR combined with CyclinD1, CUDR combined with PTEN depletion; (3) C-myc activity is crucial for the synergetic oncogenic effect of CUDR overexpression plus CyclinD1 overexpression or CUDR overexpression plus PTEN depletion.

To our knowledge, we first proved that CUDR exerts its oncogenic effect in part through the upregulation and activation of TERT and C-myc. Our present approaches provided an unequirocal evidence for critical oncogenic roles of the CUDR in liver cancer stem cells, liver stem cells and supported the notion that CUDR may be an alternative bona fide promoting factor of liver stem cells malignant transformation. However, we have fully not understood the accuracy mechanism of CUDR combined CyclinD1 and PTEN, such as, how CUDR works together CyclinD1 or PTEN? What are the partners of CUDR during genes regulation and control? In this report, we focused mainly on the view that CUDR overexpression combined with CyclinD1 overexpression or PTEN depletion promotes liver stem cells malignant by activating TERT dependent on H19 and upregulating C-myc by CTCF mediated DNA looping. In conclusions, our present findings open the possibility that targeting CUDR, CyclinD1 and upregulating PTEN might prove to be an alternative therapeutic strategy. It will produce an important implication for treatment and diagnosis of hepatocarcinoma.

## MATERIAL AND METHODS

### Human liver cancer stem cell line (HLCSC) sorting

CD133/CD44/CD24/EpCAM MicroBead Kits were purchased from Miltenyi technic (Boston, USA) and MACS^®^ Technology operation according to and the operation according to the manufacturer. In brief, centrifuge cell suspension at 300 × g for 10 minutes and. Resuspend cell pellet in 300 μL of buffer per 10^8^ total cells after aspirating supernatant completely. Add 100 μL of FcR Blocking Reagent per 10^8^ total cells and 100 μL of CD133/CD44/CD24/EpCAM MicroBeads per 10^8^ total cells. Mix well and incubate for 30 minutes in the refrigerator (2 − 8°C). Wash cells by adding 1 − 2 mL of buffer per 10^8^ cells and centrifuge at 300 × g for 10 minutes. Resuspend up to 10^8^ cells in 500 μL of buffer. Choose an appropriate MACS Column and MACS Separator according to the number of total cells and the number of CD133+/CD44+/CD24+/EpCAM+ cells. CD133+/CD44+/CD24+/EpCAM+ cells can be enriched by using MS Columns or depleted with the use of LD Columns. Place column in the magnetic field of a suitable MACS Separator. Prepare column by rinsing with the appropriate amount of buffer MS (500 μL). Apply cell suspension onto the column. Collect flow-through containing unlabeled cells. Wash column with the appropriate amount of buffer. Collect unlabeled cells that pass through and combine with the effluent from step MS (3 × 500 μl). Remove column from the separator and place it on a suitable collection tube.

The stem cells of breast cancer, lung cancer, gastric cancer and leukemia cells from Human liver cancer Huh7, human breast cancer cell line MCF7, human lung cancer cell line A549, human gastric cancer cell line SGC-7901, human leukemia cell line THP-1 by MicroBead Kits were purchased from Miltenyi technic (Boston, USA) and MACS^®^ Technology operation according to and the operation according to the manufacturer.

### Cell lines and plasmids

HLCSC cell lines were maintained in Dulbecco's modified Eagle medium (Gibco BRL Life Technologies) or Minimum Essential Medium (MEM) (Gibco BRL Life Technologies) supplemented with 10% heat-inactivated (56°C, 30 minutes) fetal bovine serum (sigma) in a humidified atmosphere of 5% CO_2_ incubator at 37°C. Plasmid pGFP-V-RS, pRFP-V-RS pCMV6-A-GFP, were purchased from Origene (Rockville, MD 20850, USA). Origene (Rockville, MD 20850, USA). pcDNA3.1-CyclinD1, pCDNA3.1-PTEN, pGFP-V-RS-CUDR, pCMV6-A-GFP-CUDR, pGFP-V-RS-H19, pGFP-V-RS-C-myc, pRFP-V-RS-PTEN and pGFP-V-RS-cyclinD1, pGFP-V-RS-TERT were prepared by ourselves. RNAi sequence: ***CUDR***: Sh1:TTCAGACTCAGCCCACTTGCACCCAAGTG;Sh2:TCTCACCAATT TCAAATCGGATCTCCTCG;Sh3:CTTTCCACAFCCTACCCCAGCCCTAT AAA;Sh4:AGCCATATGAAGACACCCTAGCTGGACGA. ***H19***:Sh1:AGCCAAGGAGCAC CTTGGACATCTGGAGT;Sh2:CTTTTGGTTACAGGACGTGGCAGCTGGTT;Sh3:ATGAATATGCTGCACTTTACAAACCACTGC;Sh4;GGCCGGGTGACTGGGCGCCGGCTGTGTGC. ***CTCF***:Sh1:CATGTGCGATTACGCCA GTGTAGAAGTCA;Sh2:AAGGTGATGCAGTCGAAGCCATTGTGGAG;Sh3:ATGGCCTTTGTGACCAGTGGAGAATTGGT;Sh4:TGTCCACTTGCGAAAGCAGCATTCCTATA. ***PTEN***:Sh1:GGTCTGAGTCGCCTGT CACCATTTCCAGG;Sh2:CTTGACCAATGGCTAAGTGAAGATGACAA;Sh3:GCAGTTCAACTTCTGTAACACCAGATGTT;Sh4:GTACAGGAATGAACCTTCTGCAACATCTT. ***TERT***:Sh1:CTGTACCAGCTCGG CGCTGCCACTCAGGC;Sh2:TTCCGCCAGGTGTCCTGCCTGAAGGAGCT;Sh3:TACGCCGAGACCAAGCACTTCCTCTACTC;Sh4:AGGCACTGTTCAGCGTGCTCAACTACGAG. ***CyclinD1***:Sh1:TTCGTGGCCTC TAAGATGAAGGAGACCAT;Sh2:TCTGTGCCACAGATGTGAAGTTCATTTCC;Sh3:TGGAACACCAGCTCCTGTGCTGCGAAGTG;Sh4:GCCATGAACTACCTGGACCGCTTCCTGTC. ***C-Myc***:Sh1:GAGGATATC TGGAAGAAATTCGAGCTGCT;Sh2:GGAAACGACGAGAACAGTTGAAACACAAA;Sh3:GAGAAGCTGGCCTCCTACCAGGCTGCGCG;Sh4:ATCATCATCCAGGACTGTATGTGGAGCGG.

### Embryonic stem (ES) cells differentiate into hepatocyte-like cell *in vitro*

Human ES cell line MEL-1 could efficiently generate definitive endoderm (DE) tissue by treating the the modified cultures with high concentrations of the TGFβ family ligand activin A (100 ng/ml, R and D, Minneapolis) for 5 days. A number of groups have generated hepatoblosts using this DE tissue as a starting material, plating the DE on matrix (e.g. collagen) to mimic the hepatic ECM and then added FGF4 (100 ng/ml, R & D) and BMP (100 ng/ml, R and D, Minneapolis) to mimic hepatic induction for 5 days (induced hepatoblasts). This is followed by some combination of insulin, transferrin, selenite (ITS,5 μg/ml, R&D, Minneapolis), HGF (20 ng/ml, R and D, Minneapolis), OSM (10 ng/ml, R and D, Minneapolis), αFGF (50 ng/ml, R and D, Minneapolis) and Dexamethasone (10^−7^M, R&D, Minneapolis) to expand the hepatoblast population and to promote hepatic maturation for 10 days (induced hepatocyte-like cells).

### Cell transfection and stable cell lines

Cells were transfected with DNA plasmids using transfast transfection reagent lipofectamine^R^ 2000 (Invitrogen) according to manufacturer's instructions. For screening stable cell lines, forty-eight hours after transfection, cells were plated in the selective medium containing G418 (1000–2000 μg/ml, Invitrogen, Ltd., U.K) or Puromycin (1–2 μg/ml, Calbiochem) for the next 4 weeks or so, and the selective media were replaced every 3 days.

### Quantitative telomerase detection

The telomerase activity was measured by using Quantitative Telomerase Detection Kit (MT3010) according to manufacturer's instructions (US Biomax, Inc). In brief, Resuspend the cell pellet in 200 μl of 1× Lysis Buffer /10^5^−10^6^ cells. Incubate the suspension on ice for 30 minutes. Spin the sample in a microcentrifuge at 12,000 × g for 30 minutes at 4°C. Transfer 160 μl of the supernatant into a fresh tube and determine the protein concentration. Mix the 2 × master mix thoroughly and dispense appropriate volumes into PCR thin-wall PCR plates. Add 1 μl of test extract, heat-inactivated extracts or template controls to the individual PCR tubes containing the master mix. PCR Initial 10 min 95°C HotActivited Tag DNA Polymerase. Activation Step is activated by this heating step 3 -step cycling: Denaturation 30s 95°C;Annealing 30 s 60°C; Extention 30 s 72°C. Cycle number 40 cycles Cycle. The PCR Quantification screen is displayed during the PCR run and presents data as they are being collected in real time. Collect the threshould cycle or CT value after cycles finished. The threshould cycle is the cycle at which a statistically significant increase in Δ Rn is first detected. Threshold is defined as the average standard deviation of Rn for the early cycles, multiplied by an adjustable factor. A standard curve was generated using the reading of the threshold (CT) of Real-Time PCR.

### Telemere length assay

Telemere length was measured using Telo TAGGG PCR ELISApuls kit (Roche) according to manufacturer's instructions. A standard curve is established by dilution of known quantities of a synthesised 84 mer oligonucleotide containing only TTAGGG repeats.

### RT-PCR

Total RNA was purified using Trizol (Invitrogen) according to manufacturer's instructions. cDNA was prepared by using oligonucleotide (dT)_17–18_, random primers, and a SuperScript First-Strand Synthesis System (Invitrogen). PCR analysis was performed under the specical conditions. β-actin was used as an internal control.

### Western blotting

The logarithmically growing cells were washed twice with ice-cold phosphate-buffered saline (PBS, Hyclone) and lysed in a RIPA lysis buffer. Cells lysates were centrifuged at 12,000 g for 20 minutes at 4°C after sonication on ice, and the supernatant were separated. After being boiled for 5–10 minutes in the presence of 2-mercaptoethanol, samples containing cells proteins were separated on a 10% sodium dodecyl sulfate-polyacrylamide gel electrophoresis (SDS-PAGE) and transferred onto a nitrocellulose membranes. Then blocked in 10% dry milk-TBST (20 mM Tris-HCl [PH 7.6], 127 mM NaCl, 0.1% Tween 20) for 1 h at 37°C. Following three washes in Tris-HCl pH 7.5 with 0.1% Tween 20, the blots were incubated with 0.2 μg/ml of antibody (appropriate dilution) overnight at 4°C. Following three washes, membranes were then incubated with secondary antibody for 60 min at 37°C or 4°C overnight in TBST. Signals were visualized by ECL.

### Co-immunoprecipitation(IP)

Cells were lysed in 1 ml of the whole-cell extract buffer A (50 mM pH7.6 Tris-HCl, 150 mMNaCl, 1%NP40, 0.1 mMEDTA, 1.0 mM DTT,0.2 mMPMSF, 0.1 mM Pepstatine, 0.1 mM Leupeptine, 0.1 mM Aproine). Five-hundred-microliter cell lysates was used in immunoprecipitation with antibody. In brief, protein was pre-cleared with 30μl protein G/A-plus agarose beads (Santa Cruz, Biotechnology, Inc. CA) for 1 hour at 4°C and the supernatant was obtained after centrifugation (5,000 rpm) at 4°C. Precleared homogenates (supernatant) were incubated with 2 μg of antibody and/or normal mouse/rabbit IgG by rotation for 4 hours at 4°C, and then the immunoprecipitates were incubated with 30μl protein G/A-plus agarose beads by rotation overnight at 4°C, and then centrifuged at 5000 rpm for 5 min at 4°C. The precipitates were washed five times × 10 min with beads wash solution (50 mM pH7.6 TrisCl, 150 mMNaCl, 0.1%NP-40, 1 mM EDTA) and then resuspended in 60μl 2 × SDS-PAGE sample loading buffer to incubate for 5–10 min at 100°C. Then Western blot was performed with a another related antibody indicated in Western blotting.

### RNA immunoprecipitation(RIP)

Cells were lysed (15 min, 0°C) in 100 mM KCl, 5 mM MgCl_2_, 10 mM HEPES [pH 7.0], 0.5% NP40, 1 mM DTT, 100 units/ml RNase OUT (Invitrogen), 400 μM vanadyl-ribonucleoside complex and protease inhibitors (Roche), clarified and stored on at − 80°C. Ribonucleoprotein particle-enriched lysates were incubated with protein A/G-plus agarose beads (Santa Cruz, Biotechnology, Inc. CA) together with antibody or normal mouse or rabbit IgG for 4 hours at 4°C. Beads were subsequently washed four times with 50 mM Tris-HCl (pH 7.0), 150 mM NaCl, 1 mM MgCl_2_, and 0.05% NP-40, and twice after addition of 1M Urea. Immunoprecipitates (IPs) were digested with proteinase K (55°C; 30′) and mRNAs were then isolated and purified. RT-PCR was performed with the primers as follows: CUDR/P1:5′-atgagtcccatcatctctcca-3′; CUDR/P2: 5′-taatgtaggtggcgatgagtt-3′.

### Super-EMSA(gel-shift)

Cells were washed and scraped in ice-cold PBS to prepare nuclei for electrophoretic gel mobility shift assay with the use of the gel shift assay system modified according to the manufacturer's instructions (Promega). In brief, consensus oligonucleotides for damage or repair DNA was biotin-labeled probe. Each binding reaction was carried out with 1 μg biotinylated dsDNA probe and 200 μg purified nuclear protein in 20 μl of binding buffer containing 0.5 mg/ml poly (dI:dC) (25 mM HEPES at PH8.0 with 50 mM KCl. 0.1% Triton X100, 2 mM MgCl2, 3 mM DTT, and 5% glycerol). Twenty-five pmol unlabeled cold DNA motifs (a 500-fold excess) were added in the competition assays. Reactions were carried out for 30 min incubation at room temperature, followed by overnight incubation at 4°C. Reaction mixtures were loaded onto 6% TBE polyacrylamide gels and separated in 0.5% × TBE at 100 v on ice until the dye front migrated two-thirds of the way to NC membranes and Western blotting for anti-biotin.

### Chromatin immunoprecipitation (CHIP) assay

Cells were cross-linked with 1% (v/v) formaldehyde (Sigma) for 10 min at room temperature and stopped with 125 mM glycine for 5 min. Crossed-linked cells were washed with phosphate-buffered saline, resuspended in lysis buffer, and sonicated for 8–10 min in a SONICS VibraCell to generate DNA fragments with an average size of 500 bp or so. Chromatin extracts were diluted 5-fold with dilution buffer, pre-cleared with Protein-A/G-Sepharose beads, and immunoprecipitated with specific antibody on Protein-A/G-Sepharose beads. After washing, elution and de-cross-linking, the ChIP DNA was detected by either traditional PCR (30 cycles) and PCR products were run on a 2% agarose gel.

### DNA methylation analysis

mthylated DNA Immunoprecipitation (MeDIP)-Dot blot-western blotting with anti-5-Methylcytosine (5-mC) and ethylation analysis by MspI plus BamHI digestion.

### *In situ* hybridization

Deparaffinization and antigen retrieval (Digest with 20 μg/ml proteinase K in pre-warmed 50 mM Tris for 10 to 20 min at 37°C). Rinse slides 5 times in distilled water. Immerse slides in ice cold 20% (v/v) acetic acid for 20 sec. Dehydrate the slides by washing for approximately 1 min each wash in 70% ethanol, 95% ethanol and 100% ethanol then air dry. Add 100 μl of hybridization solution to each slide. Incubate the slides for 1 hr in a humidified hybridization chamber at the 42°C. Under heat at 95°C for 2 min, to denature the DIG (Digoxigenin) labeled DNA probe. Drain off the hybridization solution. Add 50 μl of diluted probe per section. Incubate in the humidified hybridization chamber at 42 overnight. While incubating, the sample on the slide can be covered with a cover slip to prevent evaporation. Stringency washes: Wash 1: 50% formamide / 2 × SSC (3 × for 5 min, 37–45°C). Wash 2: 0.1–2 × SSC3 × for 5 min, 25°C to 75°C. Wash twice in MABT (maleic acid buffer containing Tween 20) for 30 min at room temperature. Dry the slides. Transfer to a humidified chamber and add 200 μl blocking buffer to each section (MABT + 2% BSA, milk or serum). Block for 1 to 2 hours, at room temperature. Drain off the blocking buffer. Add the anti-DIG antibody at the required dilution in blocking buffer. Wash slides 5 times with MABT, 10 min for each wash, at room temperature. For culture cells, following three washes, slides were then incubated with FITC-secondary antibody for 60 min at 37°C or 4°C overnight. For tissue section, following three washes, SABC-DAB staining was performed.

### Cells proliferation CCK8 assay

Cells were synchronized in G0 phase by serum deprivation and then released from growth arrest by reexposure to serum, and then cells were grown in complete medium for assay. according to the manufacturer instruction. In brief, cells at a concentration 4 × 10^3^ were seeded into 96-well culture plates in 100μl culture medium containing 10% heat-inactivated fetal calf serum (FCS). Before detected, add 10 μg/well cell proliferation reagent CCK8 and incubate for 4 hours at 37°C and 5% CO_2_. Cell growth curve was based on the corresponding the normalized values of OD450 and each point represents the mean of three independent samples.

### Colony-formation efficiency assay

5 × 10^2^ cells were plated on a 10 cm dish, the 10 ml DMEM containing 10%FBS was added into each 10 cm dish of the three replicate. Then these dishes were incubated at 37°C in humidified incubator for 10 days. Cell colonies on the dishes were stained with 1 ml of 0. 5% Crystal Violet for more than 1 hour and the colonies were counted.

### Soft agar colony formation assay

2 × 10^2^ cells were plated on a 6 well plate containing 0.5% (lower) and 0.35% (upper) double layer soft-agar. Then the 6 well plates were incubated at 37°C in humidified incubator for 21 days. The cells were fed 1–2 times per week with cell culture media (DMEM). Soft-agar colonies on the 6 well plates were stained with 0.5 ml of 0.05% Crystal Violet for more than 1 hour and the colonies were counted.

### Cells sphere formation ability assay

Cells were collected and washed to remove serum, then suspended in serum-free DMEM/F12 supplemented with 20 ng/ml human recombinant epidermal growth factor (hrEGF), 10 ng/ml human recombinant basic fibroblast growth factor (hrbFGF), 2% B27 supplement without vitamin A, 1% N2 supplement (Invitrogen, Carlsbad, CA, USA). The cells were subsequently cultured in ultra low attachment 6-well plates (Corning Inc., Corning, NY, USA) at a density of no more than 5,000 cells/well. The spheres were collected by gentle centrifugation, then dissociated with trypsin-EDTA and mechanically disrupted with a pipette. The resulting single cells were then centrifuged to remove the enzyme and re-suspended in serum-free medium allowed to re-form spheres. The spheres should be passaged every 5–8 days before they reached a diameter of 100 μm. The sphere from ten random chosen fields of at least three independent samples were counted.

### Xenograft transplantation *in vivo*

Four-weeks male athymic Balb/C mice were purchased from Shi laike company (Shanghi, China) and maintained in the Tongji animal facilities approved by the China Association for accreditation of laboratory animal care. The athymic Balb/C mouse was injected at the armpit area subcutaneously with suspension of cells in 100μl of phosphate buffered saline. The mice were observed four weeks, and then sacrificed to recover the tumors. The wet weight of each tumor was determined for each mouse. A portion of each tumor was fixed in 4% paraformaldehyde and embedded in paraffin for histological hematoxylin-eosin (HE) staining. The use of mice for this work was reviewed and approved by the institutional animal care and use committee in accordance with China national institutes of health guidelines.
